# High-quality bacterial genomes of a partial-nitritation/anammox system by an iterative hybrid assembly method

**DOI:** 10.1186/s40168-020-00937-3

**Published:** 2020-11-06

**Authors:** Lei Liu, Yulin Wang, You Che, Yiqiang Chen, Yu Xia, Ruibang Luo, Suk Hang Cheng, Chunmiao Zheng, Tong Zhang

**Affiliations:** 1 Environmental Microbiome Engineering and Biotechnology Laboratory, The University of Hong Kong, Hong Kong SAR, China; 2 State Environmental Protection Key Laboratory of Integrated Surface Water-Groundwater Pollution Control, School of Environmental Science and Engineering, Southern University of Science and Technology, Shenzhen, China; 3 School of Environmental Science and Engineering, Southern University of Science and Technology, Shenzhen, China; 4 Department of Computer Science, The University of Hong Kong, Hong Kong SAR, China; 5 Department of Chemical Pathology, The Chinese University of Hong Kong, Hong Kong SAR, China

**Keywords:** Iterative hybrid assembly, High contiguity, One-stage partial-nitritation anammox

## Abstract

**Background:**

Genome-centric approaches are widely used to investigate microbial compositions, dynamics, ecology, and interactions within various environmental systems. Hundreds or even thousands of genomes could be retrieved in a single study contributed by the cost-effective short-read sequencing and developed assembly/binning pipelines. However, conventional binning methods usually yield highly fragmented draft genomes that limit our ability to comprehensively understand these microbial communities. Thus, to leverage advantage of both the long and short reads to retrieve more complete genomes from environmental samples is a must-do task to move this direction forward.

**Results:**

Here, we used an iterative hybrid assembly (IHA) approach to reconstruct 49 metagenome-assembled genomes (MAGs), including 27 high-quality (HQ) and high-contiguity (HC) genomes with contig number ≤ 5, eight of which were circular finished genomes from a partial-nitritation anammox (PNA) reactor. These 49 recovered MAGs (43 MAGs encoding full-length rRNA, average N50 of 2.2 Mbp), represented the majority (92.3%) of the bacterial community. Moreover, the workflow retrieved HQ and HC MAGs even with an extremely low coverage (relative abundance < 0.1%). Among them, 34 MAGs could not be assigned to the genus level, indicating the novelty of the genomes retrieved using the IHA method proposed in this study. Comparative analysis of HQ MAG pairs reconstructed using two methods, i.e., hybrid and short reads only, revealed that identical genes in the MAG pairs represented 87.5% and 95.5% of the total gene inventory of hybrid and short reads only assembled MAGs, respectively. In addition, the first finished anammox genome of the genus *Ca*. *Brocadia* reconstructed revealed that there were two identical hydrazine synthase (*hzs*) genes, providing the exact gene copy number of this crucial phylomarker of anammox at the genome level.

**Conclusions:**

Our results showcased the high-quality and high-contiguity genome retrieval performance and demonstrated the feasibility of complete genome reconstruction using the IHA workflow from the enrichment system. These (near-) complete genomes provided a high resolution of the microbial community, which might help to understand the bacterial repertoire of anammox-associated systems. Combined with other validation experiments, the workflow can enable a detailed view of the anammox or other similar enrichment systems.

Video Abstract

## Background

Since the discovery of anaerobic ammonium oxidation (anammox), research in this process has increased enormously, leading to several biotechnological breakthroughs [1, 2]. One such technology, the partial-nitritation anammox (PNA) process, has been installed in more than 100 full-scale wastewater treatment plants worldwide to remove nitrogen [3]. As an important biological and biotechnological process, mechanistic insights into the anammox process are lacking and genome sequences of the relevant organisms may help in addressing this problem [4]. Recently, second-generation sequencing technology that generates short reads (SRs) has been extensively employed to characterize the microbial consortium in the ecosystem due to the high-throughput and cost-effective advantages [5].

Meanwhile, metagenome-assembled genomes (MAGs), retrieved using SRs-based assembly and binning methods [6–9], are incorporated into public genome databases at an accelerating rate with approximately 300 bacterial genomes present in 2006 [10] to more than 246 K genomes (NCBI Prokaryotes genome database) in April of 2020. However, only less than 8% of MAGs have a “complete” assembly status, while the majority of genomes are fragmented into contigs. This may limit the ability to profile a full-picture of the microbial metabolic functions in relevant studies [11, 12]. Currently, the complete genomes pertaining to anammox systems are very limited and most genomes are highly fragmented, rendering a more comprehensive understanding of the microbial community not feasible to be characterized [13–15].

Third-generation sequencing technology utilizes long reads (LRs) and was developed by both Pacific Bioscience (PacBio) and Oxford Nanopore technologies (ONT). It offers very promising prospects, resolving repeats, eliminating gaps, and capturing a more complete picture of prokaryotic genomes by significantly enhancing the contiguity of the assemblies or even recovering complete genomes [16–19]. LRs sequencing enables genomic research at an unprecedented resolution although it has a weakness of the high error rate of raw reads (PacBio ~ 13% [18], ONT ~ 10% [16]). A few LRs-based assembly workflows have been proposed [20–22], as well as hybrid assembly approaches [11, 23], which combine SRs and LRs to take advantage of the high accuracy and read length, respectively.

**Fig. 1:**
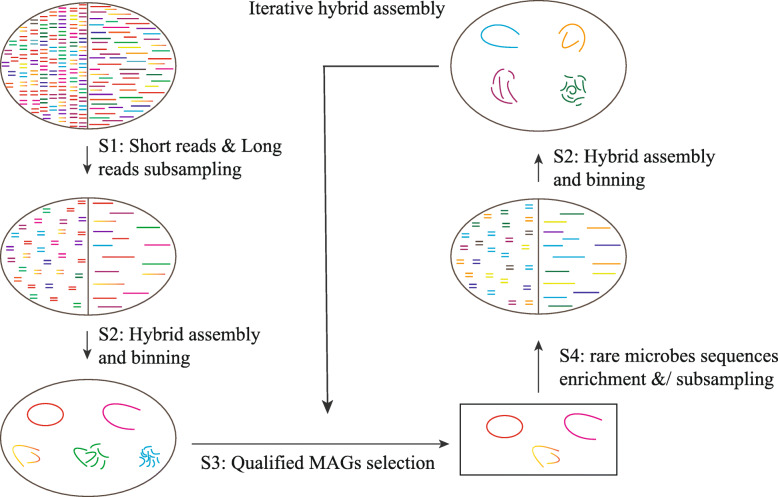
Iterative hybrid assembly workflow for enrichment ecosystems. Subsampled short reads (SRs) and long reads (LRs) are first prepared from the total sequenced dataset or from the simplified dataset that eliminates reads used for assembling qualified MAGs (step 1). SRs and LRs are then combined for hybrid assembly using Unicycler and then binning with MetaWRAP (step 2). Qualified MAGs with high quality and high contiguity were selected (step 3). SRs and LRs clusters for residual microbes in the community are obtained by taking out reads of qualified MAGs and then subsampling for the next run (step 4)

Although some studies have been endeavoring to retrieve MAGs from metagenomic samples by inclusion of long reads [11, 19, 22], how to optimally leverage the advantage of the long reads is still under investigation. Here, we initially evaluated the assembly efficiency of different assembly methods (metaSPAdes [24], megahit [7], Unicycler [23], and OPERA-MS [11]) using mock datasets. Based on the evaluation, the selected assembler (Unicycler) was integrated into the iterative hybrid assembly (IHA) workflow (Fig. 1) proposed in this study to reconstruct high-quality and high-contiguity (HQ-HC) MAGs from a PNA dataset containing 111 gigabase pairs (Gbp) Illumina and 69 Gbp Nanopore reads. The resulting high-quality MAGs served as a valuable reference and enhanced earlier investigations of this anammox ecosystem by providing a more comprehensive gene inventory and genome structure of the contained organisms [15]. The genome retrieval performance of IHA workflow showcased that this approach could be used to produce a high-resolution profile of the microbial community composition and genome structure of other enrichment systems.

## Methods

### Sampling and DNA extraction

One sludge sample was collected on November 15, 2018, from a well-operated one-stage PNA reactor under the same operating parameters as in our previous study [15]. Genomic DNA was extracted using the DNeasy PowerSoil Kit (Qiagen, Hilden, Germany) according to the manufacturer’s instructions. DNA concentration and purity were determined using a Qubit 2.0 Fluorometer (Life Technologies, Carlsbad, CA, USA) and NanoDrop 1000 spectrophotometer (Thermo Scientific, Wilmington, DE, USA) as previously described [25]. The total DNA was divided into two equal parts, with one part sequenced by Novogene Company Limited (Beijing, China) using shotgun sequencing on an Illumina HiSeq 4000 PE150 sequencer (Illumina, CA, USA) to generate 150 bp paired-end reads with a 350-bp insert size, and the other sequenced by the Chinese University of Hong Kong (Hong Kong, China) using a Nanopore PromethION sequencer (Oxford Nanopore Technology, UK).

### Illumina and PromethION sequencing and processing

Cutadapt (v1.6) [26] was used to remove adaptors from the Illumina short reads. Illumina sequencing generated SRs with an average base-quality score of ≤ 30 were filtered out, with the remaining denoted as “clean reads.” The clean reads totaled 370.8 million paired-end reads (111.2 Gbp) and were deposited into the NCBI SRA database (access number: PRJNA627084). The PromethION sequencing generated raw LRs that were base called using Albacore (v3.0.1). Only reads longer than 1 Kbp were retained, which generated 69.4 Gbp. Statistical analysis and visualization of LRs were performed using BBMap (v38.34) [27] and ggplot2 [28] (Additional file 1: Figure S1). Seqtk (v1.3) [29] was used to extract subsets of SRs or LRs from all of the reads randomly. Assembly was performed by combining SRs and LRs and by using SRs individually using corresponding assemblers. Only assembled contigs ≥ 1 Kbp were utilized for downstream analyses [30].

### Mock community datasets

The GridION LRs and Illumina bacteria isolates SRs of ZymoBIOMICS Microbial Community Standards (EVEN) (Zymo-GridION-EVEN-BB-SN) from Nicholls’s released data [16] (hereafter referred to as Mock) were downloaded to construct two mock datasets. Detailed description of the Mock community sequences can be found in Nicholls’s paper [16]. The first dataset was comprised of all Mock Illumina bacteria isolates SRs (~ 26 Gbp) and part of the GridION LRs (4 Gbp subsampling randomly), and was named the Mock dataset in this study. The second dataset called PNA-with-Spiked-Mock dataset, was constructed using 6 Gbp SRs and 22 Gbp LRs from our PNA reactor, and the Mock Illumina bacteria isolates SRs (6 Gbp, random subsampling) and GridION LRs (4 Gbp, the same as the Mock dataset), to determine whether the assembler can perform in a similar fashion with more complex systems.

To further evaluate the feasibility of Unicycler, two further complex mock datasets were used. One is the human microbiome project (HMP) mock dataset with Illumina short reads (SRR2822457) and Illumina synthetic long reads (SRR2822454) [31], the other is the GIS20 mock community, including 20 microbial species with a wide range of abundances (0.1-30%) [11].

### Assembly, binning, MAG annotations, and quality assessment

Two SR-based assemblers, megahit (v1.2.1-beta) [7] and metaSPAdes (v3.13) [24], and two hybrid assemblers, Unicycler (v0.4.7) [23] and OPERA-MS (v0.8.1) [11], were selected as candidate assemblers with the following parameters applied: megahit: --k-min 27 --k-max 127 --k-step 20; metaSPAdes: -k 21, 33, 55; Unicycler: default parameters in hybrid assembly mode; and OPERA-MS: default parameters. Additionally, based on the evaluation result of the long-read assemblers by Wick and Holt [32], Flye (v2.7, --nano-raw --meta -g 5 m) [33] was used to assemble LRs from the metagenomic data and then polished in four rounds by using racon (v1.3.1). Contigs with length less than 1 Kbp in all of the assemblies were filtered out. MAGs were retrieved by adopting the “binning” (--metabat2 [8] and --maxbin2 [34]) and “bin_refinement” modules (with parameter -c 70 -x 10) in MetaWRAP (v1.1) [9]. MAG features were identified using Prokka (v1.33) [35], with ribosomal RNA (rRNA) features determined using Barrnap (v0.9) [36] and Open Reading Frames (ORFs) were predicted using Prodigal (v2.6.3) [37], and bacterial taxonomic classifications were assigned using GTDB-Tk (v0.2.2, database release 89) [38]. The genome completeness and contamination of each MAG was evaluated using CheckM (v1.0.80) [39], utilizing single-copy core genes to evaluate genome completeness and contamination. Genomes containing a single continuous sequence are referred to as finished while high-quality MAGs means encodes for multiple (23S/16S/5S) rRNA genes, 18 tRNA genes, and the > 90%/< 5% completeness/contamination [40]. Draft-quality (DQ) MAGs were defined as MAGs having > 70% completeness, < 10% contamination, and the presence of 16S rRNA and at least 18 tRNAs. MAGs that meet all of the DQ criteria, but miss 16S rRNA were designated as low-quality (LQ) genomes. A genome bin with ≤ 5 contigs was defined as a high-contiguity (HC) genome.

### Mock community-based assembler evaluation

After MAGs were processed using four candidate assemblers, Mash (v2.1) [41] was used to identify the genome distances between MAGs and their corresponding reference genomes, and their reconstruction quality was evaluated using QUAST (v5.0.2) [42]. The assembly performance assessment indicators comprised the recovered Mock MAG count; the Mock MAGs contiguity assessment, with NGA50 (an N50-like metric) defined as the minimum aligned contig length covering 50% of the reference genome [23]; and a quality determination for each retrieved MAG, including the examination of the aligned genome fraction (AGF), genome purity (1-fraction of unaligned contig length in the reference genome) [11], gene recovery ratio (identical genes ratio accounting for mock reference genomes), and misassembly rate. The known plasmid sequences in the mock species were queried against the hybrid assembled contigs using Blast (v2.7.1) [43]. The Mock MAG observed coverage was calculated using CoverM (v0.4.0) (https://github.com/wwood/CoverM) with default parameters and the expected coverage was estimated using the sum of the sequence lengths for each bacterial isolate to normalize the MAG genome size.

### Iterative hybrid assembly using PNA data

To resolve the computational issues resulting from big metagenomic datasets during the hybrid assembly process, maximize the utilization ratio of the sequencing reads, and to ensure the reconstruction of more high-quality MAGs, an iterative hybrid assembly approach based on HQ-HC MAGs was adopted. Briefly, the IHA workflow (Fig. 1) comprised four steps: step (1) subsampling from the big dataset to lower the community complexity and data input; step (2) hybrid assembly and binning using Unicycler to hybrid-assemble SRs and LRs, followed by utilizing MetaWRAP (“initial binning” and “bin refinement” module) to retrieve MAGs; step (3) qualified MAGs selection, only high-quality and high-contiguity (HQ-HC) MAGs are reserved to avoid interference in downstream assembly; step (4) rare microbes sequences enrichment, after obtaining SRs and LRs clusters (which aligned to the above qualified MAGs), these sequences were discarded from the total reads to boost the relative abundance of the rare microbes. This process was repeated until a satisfactory result was obtained. The detailed description of the iterative hybrid assembly/binning workflow developed in this study is as follows.

Initially, 6 Gbp SRs and 22 Gbp LRs were selected for the hybrid assembly using Unicycler, with binning performed using MetaWRAP. In this study, this data combination strategy offered better binning results (Additional file 1: Figure S2), generated more HQ-HC quality MAGs, and provided a higher degree of reads utilization (SRs mapping ratio of 76%). Qualified MAGs were combined together, then all of the SRs were aligned to the combined qualified MAGs using bowtie2 (v2.3.4.3) [44] with the very-sensitive parameter. Next, unmapped bam files were generated using samtools (v1.9) [45] and unpaired reads were filtered using bedtools (v2.27.1) [46]. Identified paired SRs were then subsampled and collected for the next step. Additionally, LRs were mapped onto combined qualified MAGs using minimap2 (v2.17) [47] with parameters -x map-ont. To reduce incorrect mapping and improve the following microbes’ assembly performance, the alignments were filtered as follows: coverage ≥ 80%, similarity ≥ 80%.

All of the aligned SRs and LRs were discarded from the total reads. Then, to speed up the process, 20 million paired SRs and 1 million LRs of the remainder were subsampled using seqtk (v1.3) [29] and the above process was repeated, including the hybrid assembly, binning, and qualified MAGs selection steps. MAGs retrieved from the first loop were named H1 MAGs and the next loop H2 MAGs, with this naming convention maintained for subsequent loops. GNU parallel [48] was used to improve computing efficiency. The tool minimap2 [47] and the script *bamstats.py* [16] were used to calculate the MAG coverage in the LRs, and Bowtie2 [44] was used with the very-sensitive parameter to estimate the MAG coverage in the SRs, after being normalized against their genome sizes.

### Comparison of MAG pairs reconstructed using hybrid and short-read only strategies

All of the SRs were used to retrieve genomes by conducting the assembly using metaSPAdes [24], with kmer size parameter values of 27, 55, and 77, and then performing “binning” (--metabat2 [8] and --maxbin2 [34]) and “bin refinement” (-c 70 -x 10) in MetaWRAP (v1.1) [9]. To determine MAG differences between the two strategies, mash (v2.1) [41] was used to estimate the genome distances and find genome pairs, meaning two versions of a microbe genome with one retrieved using SRs assembly with metaSPAdes and the other by using the IHA method. Next, gene annotations were obtained using Prokka (v1.3) [35], with genes from genome pairs aligned and classified as either identical or distinct genes. Genes were considered identical only if they were present in the genome pairs with 100% amino acid identity and 100% coverage. To characterize functional differences between MAG pairs, all of the genes that were not annotated as hypothetical proteins were extracted and grouped based on their functional annotations as follows: set (I) having the same functional annotations, set (II) having more functional annotations in the hybrid MAGs than in the SRs-based MAGs, set (III) having functional annotations only in the hybrid MAGs, or set (IV) having functional annotations only in SRs-based MAGs. Finally, only higher quality versions from the MAG pairs were chosen as representative genomes in the final genome set.

## Results and discussion

### Assembler performance evaluation

**Fig. 2:**
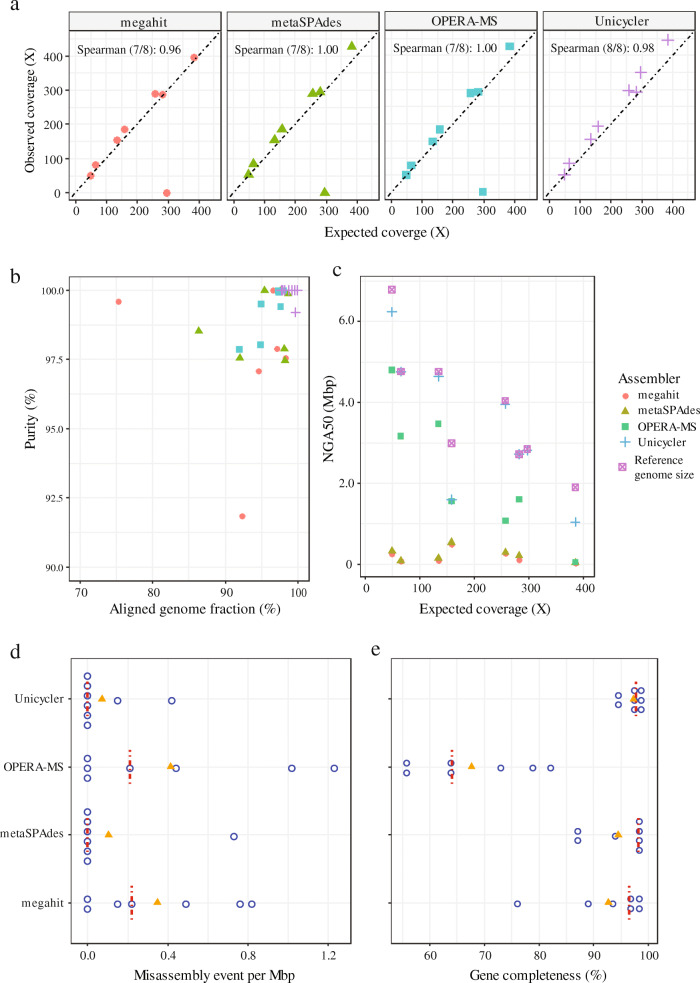
Assembler performance evaluation using the PNA-with-Spiked-Mock dataset. **a** Genome recoveries by the four assemblers, megahit (*n* = 7), metaSPAdes (*n* = 7), OPERA-MS (*n* = 7), and Unicycler (*n* = 8). **b** Reconstructed genome purity and the aligned genome fraction against the reference genome. **c** Genome continuity (NGA50) for the four assemblers as a function of genome coverage in short-read dataset. **d** Misassembly event in the assembled genome (one circle, one genome), with the orange triangle and red dashed line indicating the average and median value of the assembled genome misassembly event, respectively. **e** Gene recovery ratio and percentages of genes assembled by the four assemblers

Four assemblers (see the “Methods” section) and a more complex dataset containing part of the Mock data (PNA-with-spiked-Mock dataset), including 12 Gbp SRs and 26 Gbp LRs with an N50 of 19.5 Kbp, were used to evaluate the assembly and genome binning performance. In the dataset, the sequence of two mock yeast genomes and eight mock bacterial genomes were already known and could be used as the gold standard for evaluating accuracy of the reconstructed MAGs. Overall, seven of the eight bacteria genomes could be retrieved when using SRs-based metagenomic assemblers (megahit and metaSPAdes) and the state-of-the-art metagenomic hybrid assembler (OPERA-MS). Meanwhile, when utilizing the hybrid mode of Unicycler, all eight bacterial genomes could be recruited (average N50 = 3.476 Mbp), with six being single-contig MAGs and all eight MAGs having a NGA50 > 1 Mbp, outperforming even the assembler OPERA-MS (Fig. 2a and c). The observed SRs coverage of extracted MAGs had a very high correlation with the expected abundance of the mock reference genomes (Spearman’s coefficient range, 0.96-1.00) for the four assemblers (Fig. 2a). Additionally, the aligned genome fraction (AGF) and purity were evaluated in the paired reconstructed MAGs and in the mock reference genomes. The results demonstrated that Unicycler has the best assembly performance, with an average AGF of 98.9% and purity of 99.9% (Fig. 2b). Although MAGs reconstructed using Flye-based assemblies with high average AGF (99.42 ± 0.84%) and purity (99.79 ± 0.40%), the average completeness of retrieved MAGs was 81.64 ± 4.92%, so Flye-based MAGs were not included in this evaluation.

Notably, the NGA50s of the mock MAGs reconstructed by hybrid assemblers (OPERA-MS and Unicycler) were far better than the SRs-based assemblers (megahit and metaSPAdes). In total, OPERA-MS provided about a 17X and 12X NGA50 improvements when compared with megahit and metaSPAdes, respectively; while Unicycler even doubled the contiguity of OPERA-MS to a 29X and 22X improvement over the SRs-based assemblers with nearly no assembly errors (median and average misassembly rate of Unicycler were 0 and 0.07 per Mbp, respectively) (Fig. 2d).

Predicted genes are central to analyzing metabolic, functional, and evolutionary processes [30, 49], and thus the gene recovery ratio of each assembled MAG by using the four assemblers was further examined. The SRs-based assembler, metaSPAdes, showcased the highest median gene recovery ratio (98.2%), followed by Unicycler (97.9%). However, Unicycler had a lower recovery variation, with an average gene recovery of 97.3%, while metaSPAdes was at 94.5% (Fig. 2e). The error rates between hybrid assemblers and SRs-based assemblers were comparable, although the former showed a slightly higher mismatch and indel rates (Additional file 1: Figure S3, A and B). Similar results were obtained when using the Mock dataset that only contained pooled SRs and LRs from the mock community (see the “Methods” section and Additional file 1: Figure S4).

Unicycler also presented the feasibility to retrieve plasmid sequences. Two of the mock species contained four plasmids in their cells, which were not grouped into the corresponding MAGs successfully during the binning process. Therefore, to verify the suitability of using hybrid assemblers to assemble the plasmids, the unbinned contigs were re-examined. Unicycler was able to assemble all four plasmids (100% coverage and 100% similarity at nucleotide level) from the Mock dataset, while OPERA-MS could only assemble two of the plasmids. This finding suggests that Unicycler has stronger plasmid recovery ability. Additionally, when examining the PNA-with-Spiked-Mock dataset, Unicycler retrieved two circular plasmids, while the other assembler did not identify any plasmid.

Evaluation of assembly performance using HMP and GIS20 mock datasets, further demonstrated the feasibility of using Unicycler-based IHA strategy to retrieve MAGs from the mixed mock community datasets (Additional file 1: Figure S5 and Figure S6). Overall, LRs sequencing is appealing for genome binning and proves to be indispensable in uncovering bacterial diversity. Of the examined assemblers, Unicycler succeeded in reconstructing more genomes with a higher accuracy even though it was not initially designed for metagenomic assembly purposes [23]. Moreover, high-contiguity MAGs retrieved by hybrid assemblers can provide a more detailed genome picture and even furnish a (near-) complete gene inventory, including gene arrangements. In addition, the HQ-HC MAGs may help to address other issues, e.g., the host identification of viruses or antimicrobial resistance genes in complex bacterial communities [12, 50, 51].

### Iterative hybrid assembly for dominant and rare microbes

After evaluating assembly performance, Unicycler was chosen to reconstruct MAGs from the PNA dataset. However, Unicycler was originally developed for pure cultures or bacterial isolates [23]; thus, when attempting to process large amount of metagenomic sequencing data, the assembly performance deteriorated, with memory issues and an extended processing time. For the PNA dataset, 111.2 Gbp paired-end SRs (PE 150) and 69.4 Gbp LRs (6.9 million reads, N50 = 23 Kbp) were sequenced. To address the above issues, while leveraging the advantages of the assembler so high-quality MAGs can be obtained, a multistage sampling and iterative hybrid assembly strategy was initiated.

First, a data combination strategy was employed, with 6 Gbp SRs and 22 Gbp LRs subsampled. The reads were assembled into 328 contigs (N50 = 3.379 Mbp), with 16 of them being longer than 2.5 Mbp and seven out of the above contigs being circular (Additional file 1: Figure S7), assembly graphs were visualized using Bandage (v0.8.1) [52]. After binning, using MetaWRAP, 18 MAGs were recovered. Among them, eleven MAGs were single-contig, including seven complete circular MAGs (one anammox, two Ammonia-oxidizing bacteria (AOB), three *Planctomycetota*, one *Armatimonadota*, and one *Bacteroidota* genomes), and additional six MAGs were binned into five contigs or fewer, thus implying that this tool can provide a robust assembly efficiency. While using random subsampling for assembly and binning can recover dominant bacteria genomes within an ecosystem, other bacterial genomes might fail to be reconstructed due to low coverage and/or too much assembly/binning interference, decreasing the explanation of the complex system. The herein proposed IHA approach aims to enhance the utilization of sequencing data, improve assembly performance of both the dominant and rare microbes and decrease the runtime. Retrieval of high-quality MAGs of low-abundance microbes can be accomplished by detaching high-abundance qualified MAGs data and the runtime can be shortened by reducing the sequence data complexity and performing multistage sampling.

**Fig. 3:**
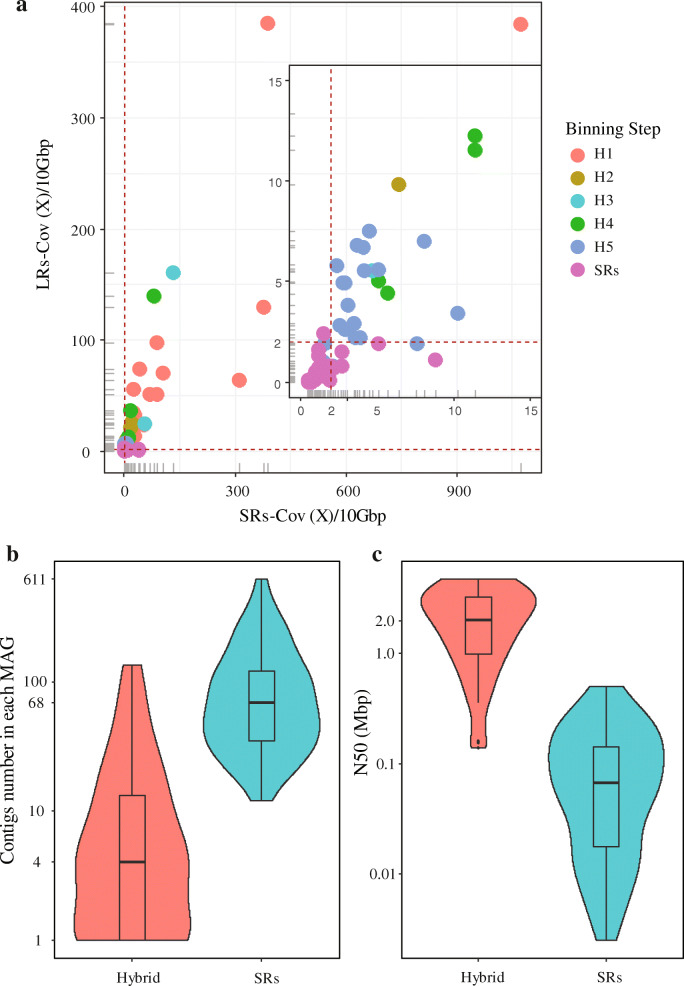
Assembly of a deep-sequenced PNA microbiome by using an iterative hybrid assembly (IHA) approach. **a** Coverage distribution of assembled MAGs after five cycles in short reads and long reads datasets that were normalized to 10 Gbp. **b**, **c** Contig numbers and N50 distributions for 47 paired MAGs assembled by using the hybrid and SR only approaches. Two MAGs only assembled by the hybrid approach were not involved in the comparison

Overall, for the assembly of the PNA anammox ecosystem, when utilizing an exclusively SRs assembly method (metaSPAdes), 85 MAGs with an average N50 of 103 Kbp (N50 = 152 Kbp for the top 55 MAGs) were reconstructed, including 10 HQ, 17 DQ, and 58 LQ MAGs that accounted for 81.7% of the SRs. For the IHA approach, after completing 5 cycles of assembly/binning, genomes from each cycle were combined and dereplicated. Fifty-five MAGs with an average N50 of 1.950 Mbp (> 12-fold improvement from the SRs-only method), including 27 HQ-HC MAGs (8 complete circular genomes; Table 1), 12 additional HQ MAGs, 13 DQ MAGs, and 3 LQ MAGs that comprised 92.6% of the total sequenced DNA (based on SRs), were retrieved. After performing de-replication of MAGs retrieved by both methods, the final genome set contained 87 MAGs (96.2%), with 49 MAGs (Additional file 2: File S1) from the IHA method and 38 from the SRs-only method (Fig. 3a).

**Table 1:** Genome features for 27 high quality and high contiguity MAGs recovered from the PNA ecosystem using the Iterative Hybrid Assembly (IHA) approach. Taxonomic assignments were identified using GTDB-Tk

MAGs ID	Status	Genome size (Mbp)	N50 (Mbp)	No. of contigs	GC	C/C (%)	Features (CDS/rRNA/tRNA)	Taxonomy
H1_BAC1		3.379	3.379	1	0.460	98.5/0.3	3138/3/40	*Bacteroidota* (family *Cyclobacteriaceae*)
H1_PLA1		3.610	3.610	1	0.700	98.9/0.0	3039/3/49	*Planctomycetota* (order *Phycisphaerales*)
H1_PLA2	Circular	3.877	3.877	1	0.633	96.6/0.0	3140/3/51	*Planctomycetota* (class *Phycisphaerae*)
H1_AMX1	Circular	3.495	3.495	1	0.425	100.0/1.6	3099/3/47	*Planctomycetota* (genus *Brocadia*)
H1_AOB3		2.842	2.842	1	0.512	100.0/0.3	2674/3/43	*Proteobacteria* (genus *Nitrosomonas*)
H1_ARM1	Circular	2.897	2.897	1	0.609	94.9/0.9	2665/3/48	*Armatimonadota* (family *Fimbriimonadaceae*)
H1_PLA3	Circular	3.887	3.887	1	0.673	96.6/0.0	3032/3/53	*Planctomycetota* (class *Phycisphaerae*)
H1_PLA4	Circular	3.841	3.841	1	0.672	97.7/0.0	3229/3/54	*Planctomycetota* (order *Phycisphaerales*)
H1_AOB1	Circular	3.279	3.279	1	0.499	99.8/0.0	3122/3/38	*Proteobacteria* (genus *Nitrosomonas*)
H1_CFX2		4.791	4.742	4	0.531	93.6/3.6	4575/3/50	*Chloroflexota* (order *Anaerolineales*)
H1_AOB2	Circular	3.217	3.217	1	0.495	99.9/0.0	3074/3/39	*Proteobacteria* (genus *Nitrosomonas*)
H1_BAC2		2.842	2.842	1	0.495	97.0/1.1	2326/6/44	*Bacteroidota* (family *Kapabacteriaceae*)
H1_PLA5		5.011	3.408	4	0.610	97.7/1.1	4106/3/52	*Planctomycetota* (class *Phycisphaerae*)
H1_PLA6		5.026	3.822	2	0.650	97.7/1.7	4074/3/51	*Planctomycetota* (class *Phycisphaerae*)
H2_CFX3		4.684	1.286	5	0.566	100.0/2.7	3904/6/49	*Chloroflexota* (class *Anaerolineae*)
H2_MYX1		9.475	3.220	5	0.698	93.9/2.6	8465/3/79	*Myxococcota* (family *Polyangiaceae*)
H2_BAC3	Circular	4.213	4.213	1	0.441	98.9/0.0	3318/3/43	*Bacteroidota* (order *Ignavibacteriales*)
H2_PRO1		3.105	3.105	1	0.686	91.0/1.5	2810/3/51	*Proteobacteria* (class *Gammaproteobacteria*)
H3_ACI1		2.971	1.982	2	0.538	96.6/2.6	2688/3/47	*Acidobacteriota* (family *Pyrinomonadaceae*)
H3_CFX4		3.148	1.455	4	0.667	93.1/1.0	3207/3/48	*Chloroflexota* (class *Dehalococcoidia*)
H3_PLA7		3.869	0.987	5	0.653	94.3/3.4	3180/3/67	*Planctomycetota*
H5_SPI1		4.129	2.456	4	0.531	96.5/0.0	4087/3/40	*Spirochaetota* (order *Leptospirales*)
H5_BAC4		3.186	2.101	3	0.388	99.5/1.7	2670/3/42	*Bacteroidota* (class *Bacteroidia*)
H5_UNCL1		4.583	2.293	3	0.545	96.6/2.7	3627/3/49	*BRC1* (species *OLB16*)
H5_CFX6		2.882	1.591	2	0.525	92.7/0.9	2714/3/48	*Chloroflexota* (order *Anaerolineales*)
H5_SPI2		3.423	2.007	4	0.346	100.0/0.0	3317/6/37	*Spirochaetota* (family *Leptospiraceae*)
H5_BAC6		3.824	2.408	4	0.348	99.4/0.0	3310/3/44	*Bacteroidota* (family *Ignavibacteriaceae*)

Note: MAGs ID is short for the phylum-level taxonomy assigned using GTDB-Tk. The red asterisk means the genome was classified using the identified 16S rRNA, which had a significant improvement in classification. The column “C/C” stands for the completeness and contamination for each reconstructed MAG evaluated by using CheckM. The “Taxonomy” column contained the phylum level assignments, as well as the highest clade level annotations so far in the bracket

As expected, MAGs with high abundance (relative abundance > 0.5% in the PNA system) have a much higher contiguity (Fig. 5b), with an average contig number of two and N50 of 3.278 Mbp. Besides, the IHA approach resulted in significantly more contiguous MAGs than the SR-based method (paired sample *t* test, *P* < 0.001). Thus, this highlighted the significance of hybrid assembly in the extension of highly fragmented contigs that are ubiquitous in SRs-based assembly and binning processes [14, 15, 53–56]. Furthermore, these results showcased that the IHA workflow can also retrieve MAGs with a very low relative abundance. For instance, there were two reconstructed MAGs (H5_UNCL2 and H5_GWC) in the final genome set that only had ~ 1.5X/10 Gbp and a relative abundance of 0.04% within the total community. For the PNA system in this study, almost all the reconstructed MAG (> 70% completeness and < 10% contamination) had a coverage of > 2X/10 Gbp in SRs and > 2X/10 Gbp in LRs (Fig. 3a), in which the unit (per 10 Gbp) means that the genome coverage of MAG was normalized to 10 Gbp.

### Comparison of MAG pairs recovered by using the two strategies

Before the de-replication of the final genome set, 26 HQ-HC MAGs (see HQ-HC MAGs in Table 1, except H1_CFX2, which was only assembled using IHA approach) and their paired MAGs from SRs-only assembled method were selected for the comparison of MAGs reconstructed by using two approaches. The 26 representative MAGs were all derived from the hybrid-assembly approach and were considered very close to complete genomes (average N50 = 2.827 Mbp, completeness = 97.22 ± 2.58% and contamination = 1.00 ± 1.08%), and thus were regarded as reference genomes herein. Despite the 26 MAG pairs from SRs based and the IHA approaches having comparable completeness and contamination levels (94.73 ± 6.14% and 0.94 ± 1.13%, respectively), the average N50 improvement of MAGs was 14-fold for the IHA versus SRs-based approach (Additional file 2: File S2). Among them, eleven MAG pairs have the identical genome completeness and contamination (average genome completeness and contamination is 97.66 ± 1.70% and 1.02 ± 1.42%, respectively); however, the average genome size of the MAGs reconstructed by the hybrid assembly and short-read only methods are 3.92 ± 0.53Mbp and 3.74 ± 0.47Mbp, respectively. This result indicated that lineage-specific single-core gene-based genome evaluation can overestimate the genome completeness of the MAGs retrieved by the SRs assembly-based method. Besides, a fragmented MAG, even with a very high completeness, can also lose portions of the genomic information due to gaps between contigs or scaffolds within each MAG. This partially explains why the 85 SRs-based MAGs recruited less total DNA than the 49 MAGs reconstructed by using the IHA approach; thus, illustrating the advantage of the hybrid method in gap bridging (Fig. 3b and c).

In many cases, predicting the functional and metabolic capabilities of a certain microorganism depends on identifying genes encoding known proteins [57–59]. Therefore, predicted genes from the MAG pairs were compared and the identical gene ratios were estimated based on the percentage of genes with 100% coverage and 100% identity (amino acid level) within the hybrid assembled representative genomes. For the 26 MAG pairs, the average identical gene ratio was 87.5 ± 8.7%; with these identical genes accounting for an average of 95.5 ± 4.6% of the gene inventory for SRs-only assembled MAGs. If only considering the 22 (out of 26) MAG pairs whose completeness and contamination met the criteria of high-quality genomes, the identical gene ratio would increase to 88.9 ± 6.5% (the identical genes accounted for 95.2 ± 4.9% of SRs-only assembled MAGs). This means that still more than 11% of the total genes could not be identified when only utilizing SRs and explained why the above SRs-only assembled MAGs had a smaller genome size when compared with the representative genomes.

When profiling the functional traits of a microbial ecosystem community or a single species, it is necessary to obtain a full picture of the functional genes within the ecosystem or within an individual microbe [50, 60]. To identify the functional genes differentiation between each MAG pair, genes annotated as a “hypothetical protein” (48.5 ± 5.3%) were removed from the total predicated genes. The weighted functional gene set distributions within the 26 pairs were profiled (Additional file 1: Figure S8), including the set I, the set II, set III, and set IV (see the “Methods” section for definitions). Here, the proportion of the set I stands for the functional gene recovery ratio of the MAGs retrieved using only SRs. The higher ratio of gene recovery, the more value of the fragmented-and-HQ MAGs assembled by SRs only. Of the above 22 MAG pairs, 92.7 ± 4.4% of the functional genes from the representative reference genomes (the hybrid genomes) were in the same functional gene set (set I), and they represented 95.7 ± 3.0% of functional genes in SRs-only assembled MAGs.

**Fig. 4:**
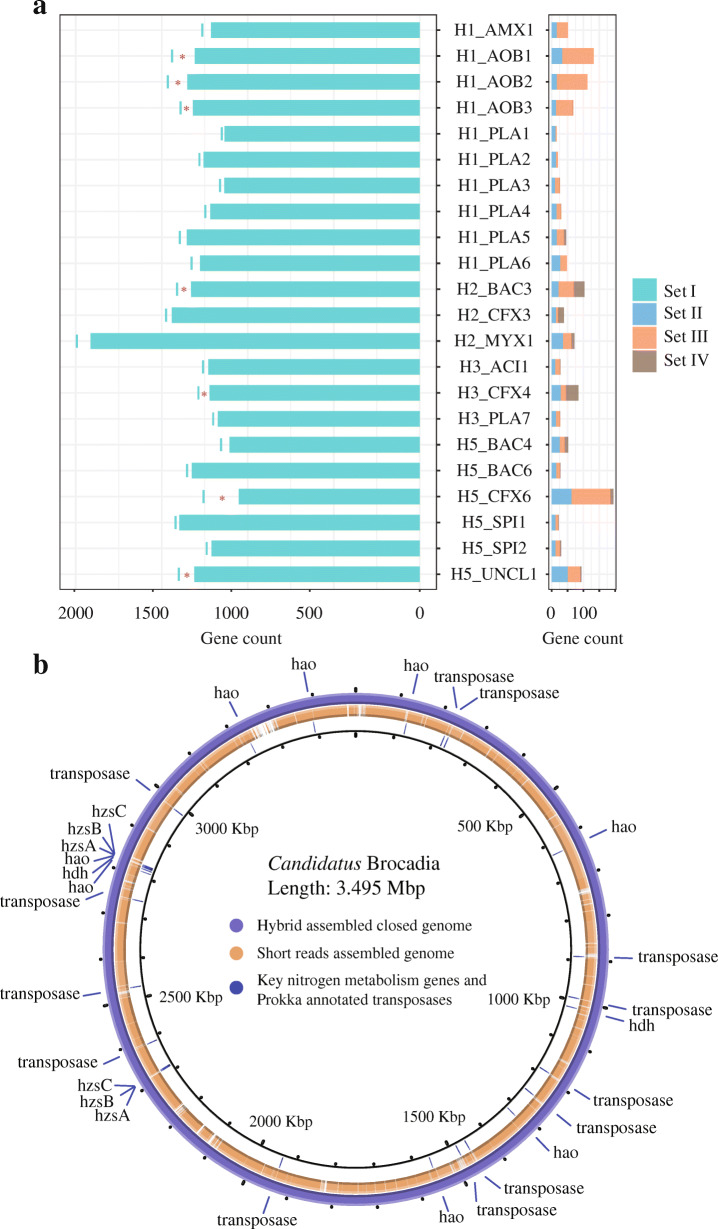
Genomes comparison retrieved by using two strategies. **a** Unweighted functional gene set distributions for 22 paired MAGs. The red asterisk indicates that genes in sets II and III accounted for more than 3% of the total functional genes from the representative genome. **b** Circular closed genome of Ca. *Brocadia*. The outermost ring stands for the closed genome reconstructed by hybrid assembly approach. The middle ring stands for the MAG reconstructed by short reads-only assembly method, which was mapped to the hybrid assembled genome. The inner ring indicated the key nitrogen metabolism gene associated with the anammox process and transposases predicted by Prokka

The metabolic potential of the microbial community or a given bacterial genome always depends on the detection of functional genes associated with metabolic pathways [59]. Thus, the unweighted functional gene set distribution was examined to underscore the significance of gene existence/nonexistence in metabolic characterization (Fig. 4a). The results were more positive with a functional gene recovery ratio of 95.8 ± 3.4% in IHA MAGs, which represents 97.5 ± 1.9% of functional genes in MAGs identified using SRs-only assembled method. For set II, 1.7 ± 1.0% of the functional genes assembled using hybrid MAGs had a higher copy number than their paired counterparts, while 2.5 ± 2.8% of functional genes (set III) were only identified in the IHA MAGs. Taken together, the comparison results from the MAG pairs demonstrate that even HQ MAGs that are assembled by using conventional SRs can miss genes to a degree, which may underestimate the metabolic potential of the microbial community and reduce the capability of deciphering their biological roles within the ecosystem. The existence of multi-copy genes or repeat sequences (especially with a high copy number) in bacterial genomes can further hinder the binning recovery performance when only SRs were used [17, 61].

### IHA produces high-resolution microbial community and genome structure profiles

**Fig. 5:**
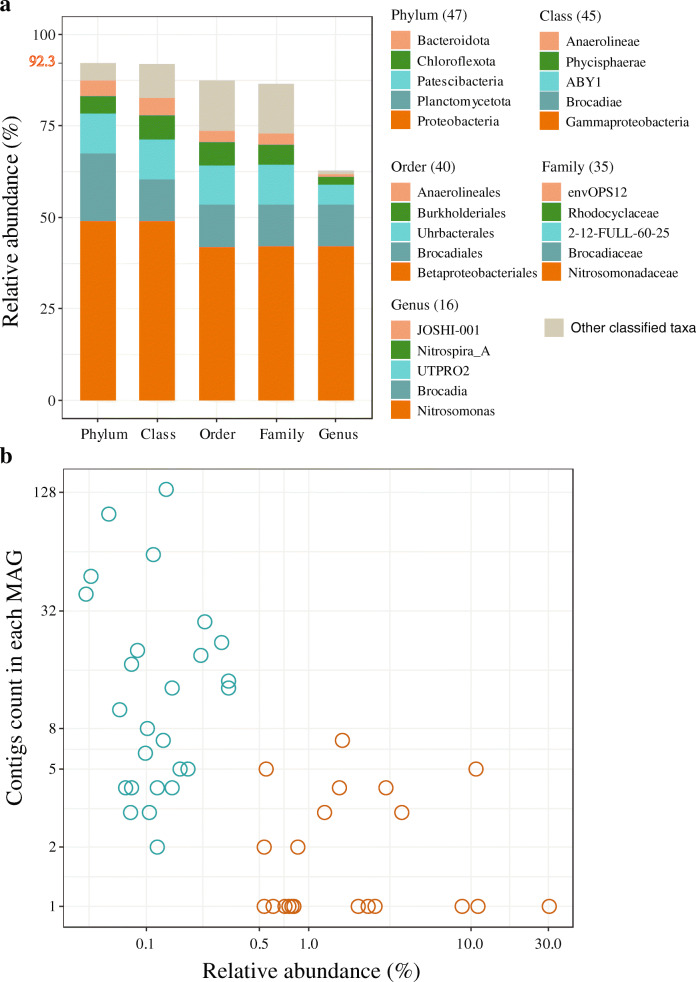
Community composition of the 49 MAGs assembled by using the iterative hybrid assembly (IHA) approach. **a** Taxonomic composition of the assembled MAGs, with only the top four most frequently observed lineages in the PNA system displayed and the remaining taxon grouped into other classified taxa. **b** Contig counts for each assembled MAG and their relative abundance in the community. The blue and brown circles indicated MAGs with relative abundance < 0.5% and > 0.5% in the community, respectively

When examining the PNA system, 49 MAGs were assembled (after de-replication) using the IHA approach, and their most likely taxonomic lineages were classified using GTDB-Tk [38], a toolkit based on a genome taxonomy database. Of these 49 MAGs that represent 92.3% of the microbial community, 33 MAGs (30.2% of total SRs) could not be assigned to a known genus and 14 MAGs (5.8% of total SRs) were unable to be classified at the family level (Fig. 5a). Furthermore, no MAGs could be assigned at the species level (Additional file 2: File S1). Overall, the annotation results suggested that a large number of bacteria within the PNA system are not well-characterized microorganisms.

Within these hybrid-assembled MAGs (*n* = 49), full-length 16S/23S rRNA operons were identified with a total of 54 copies, in which 5 of the MAGs had a 16S/23S rRNA gene copy number of 2. In contrast, for the 85 SRs-based MAG assemblies, complete 16S/23S rRNA operons could only be found in 17 fragmented MAGs, where no multi-copy of 16S/23S rRNA had been assembled and binned successfully. These findings imply that there is a deficiency in the SRs-based assembled MAGs regarding resolving repeat elements, consistent with previous studies [19, 62]. When examining microbial ecology, 16S rRNA-based taxonomic profiling is the most widely used [63, 64], and it has generated various comprehensive phylogenetic databases [65–67]. To determine whether more detailed MAG taxonomic assignments can be derived, the identified 16S rRNAs from our newly reconstructed MAGs were further aligned to the Silva 132 database [66], but little improvement was noted. Both approaches showed concordance with each other, suggesting that the genome-based taxonomic assignments were sufficient to cover the MAG lineages within the PNA system. Microorganisms belonging to the phyla *Bacteroidota*, *Chloroflexota*, *Proteobacteria*, and *Planctomycetota* are always identified in anammox-related ecosystems [13–15, 68]. The newly recovered (near-) complete MAGs will contribute to a further characterization of the bacterial repertoire of anammox-associated systems, as well as microbial ecology studies integrated with other technologies.

In addition to examining phylogenetic gene operons, other key functional genes (or gene clusters) associated with nitrogen cycling within the PNA system were also evaluated (Additional file 2: File S3). These genes included ammonia monooxygenase (*amo*), hydrazine synthase (*hzs*), and hydrazine dehydrogenase (*hdh*), and they were evaluated, specifically in three complete circular genomes (the functional group in the PNA system; H1_AMX1, H1_AOB1, and H1_AOB2). The H1_AMX1 genome (3.49 Mb), to the best of our knowledge, is the first complete genome for the genus *Candidatus* Brocadia, thus providing full insight into the gene structure and function for this genus. This genome contains a long element repeat (length > 2,100 bp) with 13 copies identified in the circular genome, which might explain why only highly fragmented MAGs had been recovered for this genus previously [14, 15] (Fig. 4b).

The *hzs* genes are encoded by a gene cluster containing three subunits—*hzs* subunit alpha (*hzs*A), *hzs* subunit beta *(hzs*B), and *hzs* subunit gamma (*hzs*C)—that is responsible for the intermediate production of hydrazine using ammonium and hydroxylamine in the genus Ca. *Brocadia* [69], and they have been reported to be highly expressed in meta-transcriptomic and meta-proteomic studies [14, 15]. As a unique functional and phylomarker gene, the *hzs*A gene is commonly applied for identification and quantification within an anammox community [70], and the exact copy number of *hzs*ABC genes can aid in determining an absolute quantification. Consistent with other identified genus of anammox bacteria [17, 71–73], two almost identical (overall nucleotide similarity = 100% and coverage = 100%, with only 1 mismatch in the *hzs*B gene) *hzs*ABC gene clusters were acquired when examining the circular genome of Ca. *Brocadia* (H1_AMX1) (Fig. 4b). This finding suggests that the copy number of this important phylogenetic marker should be considered when quantitative analysis is based on *hzs* genes [73]. When utilizing the SRs-based assembled MAG, no *hzs*ABC gene clusters could be identified, but one contig that only contained the three subunits of the *hzs* genes had been detected in the assembled contigs which had undergone genome binning by MetaWRAP. This result suggests that the contig harboring the *hzs*ABC gene cluster was filtered incorrectly from the genome assemblies of Ca. *Brocadia*, mostly due to uneven coverage. In general, short contigs/scaffolds might be classified into an incorrect MAG or directed away from a reconstructed bin as they might produce unreliable and/or noisy signatures for binning. Thus sometimes, the subsequent daunting genome manual curation that requires biological perspectives is essential for SRs-only assembled MAGs. This propensity further highlights the value of utilizing LRs when resolving these repeat regions and the significance of employing the iterative hybrid assembly (IHA) approach.

Another key functional gene associated with hydrazine metabolism and the anammox process in the complete Ca. *Brocadia* genome, *hdh* was identified with a copy number of two. However, no genes encoding nitrite reductase (*nir*S or *nir*K) could be found in the genome. Furthermore, one out of eight determined hydroxylamine oxidoreductase (*hao*) genes had a high homology (87% amino acid similarity) to the gene *kustc*1061, which has been described as a key enzyme catalyzing the reaction of hydroxylamine to nitric oxide [69]. These findings provide genome sequence evidence of a novel pathway to generate nitric oxide from hydroxylamine in the genus Ca. *Brocadia*.

Notably, nitrate reductase, which facilitates the conversion of nitrate to nitrite, was the most common nitrogen marker gene in the PNA system, with a presence in more than half of the hybrid-assembled MAGs (*n* = 29), and contributes to the previously proposed nitrite loop [13, 74]. Although no cytochrome cd1 nitrite reductase (*nir*S) or copper-containing nitrite reductase (*nir*K) enzymes could be found in the genome of the predominant anammox bacteria (H1_AMX1), another gene that annotated as *hao* in H1_AMX1 had a high similarity (83% amino acid similarity) with *kustc*0458, a likely candidate to reduce nitrite to nitric oxide [75]. Additionally, the widespread presence of nitrite reductase (*nir*K or *nir*S gene were identified in 20 MAGs) may contribute to substrate (nitric oxide, NO) generation for the anammox process, while also providing detoxification and improving the nitrogen removal efficiency in the system [13, 74]. However, the exchangeable intermediates or cross-feeding in the microbial community should be explored and validated by using other technologies, e.g., isotope-pairing experiments [76]. The source for NO also still remains unclear as it could be generated by both or either of the dominant anammox bacteria, and neighboring community members requires further investigation.

Additionally, nitrous oxide reductase (*nos*Z) was identified in 17 MAGs, which is the second most widespread nitrogen marker gene in the PNA system. These MAGs were affiliated with a broad range of phyla, including *Bacteroidota* (*n* = 6), *Chloroflexota* (*n* = 3), *Myxococcota* (*n* = 2), *Proteobacteria* (*n* = 2), *Acidobacteriota* (*n* = 1), *Planctomycetota* (*n* = 1), *Verrucomicrobiota* (*n* = 1), and *Spirochaetota* (*n* = 1). When combining these findings with previous metatranscriptomic evidence [15], it would suggest that the anammox-related microbes might contribute to mitigating the emission of potent greenhouse gasses. In addition to the anammox (*n* = 3) and nitrite-oxidizing bacteria (NOB, *n* = 1), bacteria from the *Planctomycetota* (*n* = 5), *Armatimonadota* (*n* = 1), *Chloroflexota* (*n* = 1), *Proteobacteria* (*n* = 2), and *Verrucomicrobiota* (*n* = 1) phyla, showed a potential capability of oxidizing nitrite to nitrate, thus illustrating the benefits of high-contiguity MAGs that capture a high-resolution genome structure rather than fragmented MAG drafts [13]. Meanwhile, consistent with previous findings [13], no single reconstructed MAG encoded all of the genes to complete the full denitrification process.

### Limitations and future perspectives

Genome sequences only show the metabolic potential, the proposed cross feeding in the system or other functional speculation of certain genes were all based on the retrieved genome sequences. To validate the hypothesis of these metabolic potentials, validation experiments, and multi-omics, e.g., metatranscriptomics, metaprotomics, and metabolomics should be integrated in the future studies.

The IHA workflow developed in the present study has presented the capability to reconstruct (near-) complete and accurate MAGs and improved the interpretation of the microbial community in the ecosystem. The premise of the IHA workflow is that HQ-HC MAGs (especially those with a relatively high abundance) could be retrieved in the first hybrid assembly cycle. So we recommend the approach to be applied to enrichment systems, rather than the high-complexity samples due to the algorithms and assumptions of both Unicycler and SPAdes. When the iterative strategy was applied to resolve a highly complex metagenomics dataset, a more suitable assembler should be considered. With the increasing high-throughput of Nanopore sequencers and improving accuracy of long reads, hybrid long-read assemblies approach, e.g., Trycycler + polishing (https://github.com/rrwick/Trycycler) and long-read assemblers, e.g., Flye, might help to resolve genomes from more complex systems.

## Conclusions

Currently, SRs-based MAG assemblies are still the largest source of genome drafts and provide access to complex microbial communities across various environmental niches [49, 53–56, 77]. However, the highly fragmented MAGs that arise from this sequencing technology hinder our full understanding of microbial metabolic potentials. Herein, the proposed IHA workflow improved both HQ and HC MAG reconstruction, even in cases with an extremely low relative abundance (< 0.1%). This approach enabled a more complete examination of the PNA reactor, an intermediate-complexity ecosystem, by leveraging the advantages of LRs that are generated from Oxford Nanopore sequencing technology.

Furthermore, this work mainly focused on reconstructing HQ genomes, and retrieved 39 HQ MAGs (accounting for 80.6% DNA SRs). The majority (*n* = 65) of the newly reconstructed 87 MAGs, including 20 HQ and HC MAGs, could not be assigned at the genus level, thus indicating that a substantial fraction of anammox associated microorganisms are poorly characterized. Reconstructed HQ and HC MAGs are imperative when utilizing a genome-centric approach to study microbial ecology and interactions. Therefore, the 27 HQ and HC MAGs identified herein can contribute to building a more comprehensive database of anammox associated microorganisms to better aid in deciphering their biological traits.

## Code availability

The Iterative Hybrid Assembly (IHA) workflow is available on the GitHub page: https://github.com/Hydro3639/Iterative-Hybrid-Assembly-for-enrichment-system.

## Supplementary Information

**Additional file 1: Figure S1.** Length distribution of long reads (LRs) generated on Oxford Nanopore PromethION platform. **Figure S2.** Initial binning results of PNA dataset using various short reads and long reads combination strategies. The short reads (SRs) and long reads (LRs) were hybrid assembled by Unicycler with parameter --min_fasta_length 1000 and then was binned by MetaWRAP (‘initial binning’ and ‘bin refinement’ modules with parameter -x 70 -c 10). **Figure S3.** Mismatches and indels rates of four assemblers, evaluated using PNA-with-Spiked-Mock dataset. (A), mismatches rate (B), indels rate. Orange triangle and red dashed line stand for the average and median value of the MAGs misassembly, respectively. The circle with red asterisk stands for a genome with mismatch rate more than 1000/Mbp. **Figure S4.** Assembler performance evaluation using Mock dataset, in which only contained a pool of SRs and LRs from mock community. Recovered MAGs number of different assemblers: megahit (6), metaspades (5), OPERA-MS (8), Unicycler (7). (A), Genomes recovery (B), Genome fraction vs genome purity (C), Genome continuity (D), Misassembly event occurred in assembled MAGs (E), Mismatches rates (F), Indels rates (G), gene recovery ratio. **Figure S5.** Assembler performance evaluation using the HMP dataset. Three cycles were performed using Unicyler-based IHA method. Only MAGs meeting the criteria (completeness > 70% and contamination < 10%) have been kept for downstream evaluation. (A-I) different evaluation indicators. Numbers in the legend indicate the MAG number retrieved using different assemblers. For B, the NGA50 improvement in each MAG pair was calculated by using NGA50 of the MAG obtained from IHA approach compared with megahit and metaSPAdes. **Figure S6.** Assembler performance evaluation using the GIS20 dataset. Three cycles were performed using Unicyler-based IHA method. Only MAGs meeting the criteria (completeness > 70% and contamination < 10%) have been kept for downstream evaluation. (A-I) different evaluation indicators. Numbers in the legend indicate the MAG number retrieved using different assemblers. For B, the NGA50 improvement in each MAG pair was calculated by using NGA50 of the MAG obtained from IHA approach compared with megahit and metaSPAdes. **Figure S7.** Initial binning result of PNA dataset of using 6 Gbp short reads (SRs) and 22 Gbp long reds (LRs). The figure was visualized by Bandage (v0.8.1). The number indicates the contig name, the blank circle stands for the single-contig MAG and the circle with colors means the circular sing-contig MAG. **Figure S8.** Weighted functional gene sets distribution in 22 MAG pairs. Red asterisk stands for the genes in Set II and Set III were accounted more than 3% of total functional genes of the representative genome.

**Additional file 2: File S1.** Genome information of 49 MAGs reconstructed by using IHA workflow (See Additional file 2: File S1). **File S2.** Comparison of 26 MAG pairs reconstructed using hybrid and short reads only assembly approaches. (See Additional file 2: File S2). **File S3.** Nitrogen related marker genes in the PNA system. (See Additional file 2: File S3).

## Data Availability

The raw nucleotide sequence data (both Illumina and Nanopore) used in the present study has been deposited in the NCBI database under project ID PRJNA627084.

## Abbreviations

PNA: Partial-nitritation/anammox
IHA: Iterative hybrid assembly
MAG: Metagenome-assembled genome
HQ: High-quality
HC: High-contiguity
*hzs*: Hydrazine synthase
anammox: Anaerobic ammonium oxidation
SRs: Short reads
LRs: Long reads
*hdh*: Hydrazine dehydrogenase
*hao*: Hydroxylamine oxidoreductase
*nos*Z: Nitrous oxide reductase
*nir*K: Copper-containing nitrite reductase
*nir*S: Cytochrome cd1 nitrite reductase
*amo*: Ammonia monooxygenase

## References

[1] van der Star WR, Abma WR, Blommers D, Mulder JW, Tokutomi T, Strous M, Picioreanu C, van Loosdrecht MC (2007). Startup of reactors for anoxic ammonium oxidation: experiences from the first full-scale anammox reactor in Rotterdam. Water Res.

[2] Wang Q (2017). A roadmap for achieving energy-positive sewage treatment based on sludge treatment using free ammonia. ACS Sustainable Chem Eng.

[3] Lackner S, Gilbert EM, Vlaeminck SE, Joss A, Horn H, van Loosdrecht MC (2014). Full-scale partial nitritation/anammox experiences--an application survey. Water Res.

[4] Kartal B, de Almeida NM, Maalcke WJ (2013). Op den Camp HJ, Jetten MS, Keltjens JT: How to make a living from anaerobic ammonium oxidation. FEMS Microbiol Rev.

[5] Bhattacharjee AS, Wu S, Lawson CE, Jetten MSM, Kapoor V, Domingo JWS, McMahon KD, Noguera DR, Goel R (2017). Whole-community metagenomics in two different anammox configurations: process performance and community structure. Environ Sci Technol.

[6] Peng Y, Leung HC, Yiu SM, Chin FY (2012). IDBA-UD: a de novo assembler for single-cell and metagenomic sequencing data with highly uneven depth. Bioinformatics.

[7] Li D, Luo R, Liu CM, Leung CM, Ting HF, Sadakane K, Yamashita H, Lam TW (2016). MEGAHIT v1.0: a fast and scalable metagenome assembler driven by advanced methodologies and community practices. Methods.

[8] Kang DD, Froula J, Egan R, Wang Z (2015). MetaBAT, an efficient tool for accurately reconstructing single genomes from complex microbial communities. PeerJ.

[9] Uritskiy GV, DiRuggiero J, Taylor J (2018). MetaWRAP-a flexible pipeline for genome-resolved metagenomic data analysis. Microbiome.

[10] Land M, Hauser L, Jun SR, Nookaew I, Leuze MR, Ahn TH, Karpinets T, Lund O, Kora G, Wassenaar T (2015). Insights from 20 years of bacterial genome sequencing. Funct Integr Genomics.

[11] Bertrand D, Shaw J, Kalathiyappan M, Ng AHQ, Kumar MS, Li C, Dvornicic M, Soldo JP, Koh JY, Tong C (2019). Hybrid metagenomic assembly enables high-resolution analysis of resistance determinants and mobile elements in human microbiomes. Nat Biotechnol.

[12] Warwick-Dugdale J, Solonenko N, Moore K, Chittick L, Gregory AC, Allen MJ, Sullivan MB, Temperton B (2019). Long-read viral metagenomics captures abundant and microdiverse viral populations and their niche-defining genomic islands. PeerJ.

[13] Speth DR, In’t Zandt MH, Guerrero-Cruz S, Dutilh BE, Jetten MS (2016). Genome-based microbial ecology of anammox granules in a full-scale wastewater treatment system. Nat Commun.

[14] Lawson CE, Wu S, Bhattacharjee AS, Hamilton JJ, McMahon KD, Goel R, Noguera DR (2017). Metabolic network analysis reveals microbial community interactions in anammox granules. Nat Commun.

[15] Wang Y, Niu Q, Zhang X, Liu L, Wang Y, Chen Y, Negi M, Figeys D, Li YY, Zhang T (2019). Exploring the effects of operational mode and microbial interactions on bacterial community assembly in a one-stage partial-nitritation anammox reactor using integrated multi-omics. Microbiome.

[16] Nicholls SM, Quick JC, Tang S, Loman NJ (2019). Ultra-deep, long-read nanopore sequencing of mock microbial community standards. Gigascience.

[17] Frank J, Lucker S, Vossen R, Jetten MSM, Hall RJ, den Camp HJM O, Anvar SY (2018). Resolving the complete genome of Kuenenia stuttgartiensis from a membrane bioreactor enrichment using Single-Molecule Real-Time sequencing. Sci Rep.

[18] van Dijk EL, Jaszczyszyn Y, Naquin D, Thermes C (2018). The third revolution in sequencing technology. Trends Genet.

[19] Overholt WA, Holzer M, Geesink P, Diezel C, Marz M, Kusel K. Inclusion of Oxford Nanopore long reads improves all microbial and viral metagenomeassembled genomes from a complex aquifer system. Environ Microbiol. 2020;22:4000-13. 10.1111/1462-2920.15186 32761733

[20] Lin Y, Yuan J, Kolmogorov M, Shen MW, Chaisson M, Pevzner PA (2016). Assembly of long error-prone reads using de Bruijn graphs. Proc Natl Acad Sci U S A.

[21] Li H (2016). Minimap and miniasm: fast mapping and de novo assembly for noisy long sequences. Bioinformatics.

[22] Moss EL, Maghini DG, Bhatt AS (2020). Complete, closed bacterial genomes from microbiomes using nanopore sequencing. Nat Biotechnol.

[23] Wick RR, Judd LM, Gorrie CL, Holt KE (2017). Unicycler: resolving bacterial genome assemblies from short and long sequencing reads. PLoS Comput Biol.

[24] Nurk S, Meleshko D, Korobeynikov A, Pevzner PA (2017). metaSPAdes: a new versatile metagenomic assembler. Genome Res.

[25] Che Y, Xia Y, Liu L, Li AD, Yang Y, Zhang T (2019). Mobile antibiotic resistome in wastewater treatment plants revealed by Nanopore metagenomic sequencing. Microbiome.

[26] Martin M (2011). Cutadapt removes adapter sequences from high-throughput sequencing reads. EMBnet J.

[27] Bushnell B (2014). BBMap: a fast, accurate, splice-aware aligner.

[28] Ginestet C (2011). ggplot2: elegant graphics for data analysis. J Royal Stat Soc.

[29] Li H (2013). Seqtk: a fast and lightweight tool for processing FASTA or FASTQ sequences. V1.

[30] Wang Y, Ma L, Mao Y, Jiang X, Xia Y, Yu K, Li B, Zhang T (2017). Comammox in drinking water systems. Water Res.

[31] Kuleshov V, Jiang C, Zhou W, Jahanbani F, Batzoglou S, Snyder M (2016). Synthetic long-read sequencing reveals intraspecies diversity in the human microbiome. Nat Biotechnol.

[32] Wick RR, Holt KE (2019). Benchmarking of long-read assemblers for prokaryote whole genome sequencing. F1000Res.

[33] Kolmogorov M, Yuan J, Lin Y, Pevzner PA (2019). Assembly of long, error-prone reads using repeat graphs. Nat Biotechnol.

[34] Wu Y-W, Simmons BA, Singer SW (2015). MaxBin 2.0: an automated binning algorithm to recover genomes from multiple metagenomic datasets. Bioinformatics.

[35] Seemann T (2014). Prokka: rapid prokaryotic genome annotation. Bioinformatics.

[36] Seemann T (2013). barrnap 0.7: rapid ribosomal RNA prediction.

[37] Hyatt D, Chen G-L, LoCascio PF, Land ML, Larimer FW, Hauser LJ (2010). Prodigal: prokaryotic gene recognition and translation initiation site identification. BMC Bioinformatics.

[38] Chaumeil PA, Mussig AJ, Hugenholtz P, Parks DH. GTDB-Tk: a toolkit to classify genomes with the Genome Taxonomy Database. Bioinformatics. 2020;36:1925–7. 10.1093/bioinformatics/btz848 PMC7703759 31730192

[39] Parks DH, Imelfort M, Skennerton CT, Hugenholtz P, Tyson GW (2015). CheckM: assessing the quality of microbial genomes recovered from isolates, single cells, and metagenomes. Genome Res.

[40] Bowers RM, Kyrpides NC, Stepanauskas R, Harmon-Smith M, Doud D, Reddy TBK, Schulz F, Jarett J, Rivers AR, Eloe-Fadrosh EA (2017). Minimum information about a single amplified genome (MISAG) and a metagenome-assembled genome (MIMAG) of bacteria and archaea. Nat Biotechnol.

[41] Ondov BD, Treangen TJ, Melsted P, Mallonee AB, Bergman NH, Koren S, Phillippy AM (2016). Mash: fast genome and metagenome distance estimation using MinHash. Genome Biol.

[42] Gurevich A, Saveliev V, Vyahhi N, Tesler G (2013). QUAST: quality assessment tool for genome assemblies. Bioinformatics.

[43] Mount DW (2007). Using the basic local alignment search tool (BLAST). Cold Spring Harbor Protocols.

[44] Langmead B, Salzberg SL (2012). Fast gapped-read alignment with Bowtie 2. Nat Methods.

[45] Li H, Handsaker B, Wysoker A, Fennell T, Ruan J, Homer N, Marth G, Abecasis G, Durbin R (2009). Genome project data processing S: the sequence alignment/map format and SAMtools. Bioinformatics.

[46] Quinlan AR, Hall IM (2010). BEDTools: a flexible suite of utilities for comparing genomic features. Bioinformatics.

[47] Li H (2018). Minimap2: pairwise alignment for nucleotide sequences. Bioinformatics.

[48] Tange O (2011). Gnu parallel-the command-line power tool. The USENIX Magazine.

[49] Quince C, Shaiber A, Esen OC, Lee ST, Rappe MS, McLellan SL, Lucker S, Eren AM, Delmont TO (2018). Nitrogen-fixing populations of Planctomycetes and Proteobacteria are abundant in surface ocean metagenomes. Nat Microbiol.

[50] Bickhart DM, Watson M, Koren S, Panke-Buisse K, Cersosimo LM, Press MO, Van Tassell CP, Van Kessel JAS, Haley BJ, Kim SW (2019). Assignment of virus and antimicrobial resistance genes to microbial hosts in a complex microbial community by combined long-read assembly and proximity ligation. Genome Biol.

[51] Kranz A, Vogel A, Degner U, Kiefler I, Bott M, Usadel B, Polen T (2017). High precision genome sequencing of engineered Gluconobacter oxydans 621H by combining long nanopore and short accurate Illumina reads. J Biotechnol.

[52] Wick RR, Schultz MB, Zobel J, Holt KE (2015). Bandage: interactive visualization of de novo genome assemblies. Bioinformatics.

[53] Stewart RD, Auffret MD, Warr A, Wiser AH, Press MO, Langford KW, Liachko I, Snelling TJ, Dewhurst RJ, Walker AW (2018). Assembly of 913 microbial genomes from metagenomic sequencing of the cow rumen. Nat Commun.

[54] Almeida A, Mitchell AL, Boland M, Forster SC, Gloor GB, Tarkowska A, Lawley TD, Finn RD (2019). A new genomic blueprint of the human gut microbiota. Nature.

[55] Woodcroft BJ, Singleton CM, Boyd JA, Evans PN, Emerson JB, Zayed AAF, Hoelzle RD, McCalley CK, Hodgkins SB, Lamberton TO (2018). Genome-centric view of carbon processing in thawing permafrost. Nature.

[56] Probst AJ, Ladd B, Jarett JK, Geller-McGrath DE, Sieber CMK, Emerson JB, Anantharaman K, Thomas BC, Malmstrom RR, Stieglmeier M (2018). Differential depth distribution of microbial function and putative symbionts through sediment-hosted aquifers in the deep terrestrial subsurface. Nat Microbiol.

[57] Cirulli ET (2016). The increasing importance of gene-based analyses. PLoS Genet.

[58] Kits KD, Sedlacek CJ, Lebedeva EV, Han P, Bulaev A, Pjevac P, Daebeler A, Romano S, Albertsen M, Stein LY (2017). Kinetic analysis of a complete nitrifier reveals an oligotrophic lifestyle. Nature.

[59] Speth DR, Orphan VJ (2018). Metabolic marker gene mining provides insight in global mcrA diversity and, coupled with targeted genome reconstruction, sheds further light on metabolic potential of the Methanomassiliicoccales. PeerJ.

[60] van Kessel MA, Speth DR, Albertsen M, Nielsen PH (2015). Op den Camp HJ, Kartal B, Jetten MS, Lucker S: Complete nitrification by a single microorganism. Nature.

[61] Chen L-X, Anantharaman K, Shaiber A, Eren AM, Banfield JF (2019). Accurate and complete genomes from metagenomes.

[62] Yuan C, Lei J, Cole J, Sun Y (2015). Reconstructing 16S rRNA genes in metagenomic data. Bioinformatics.

[63] Ortiz-Estrada ÁM, Gollas-Galván T, Martínez-Córdova LR, Martínez-Porchas M (2019). Predictive functional profiles using metagenomic 16S rRNA data: a novel approach to understanding the microbial ecology of aquaculture systems. Reviews in Aquaculture.

[64] Wu L, Ning D, Zhang B, Li Y, Zhang P, Shan X, Zhang Q, Brown MR, Li Z, Van Nostrand JD (2019). Global diversity and biogeography of bacterial communities in wastewater treatment plants. Nat Microbiol.

[65] McDonald D, Price MN, Goodrich J, Nawrocki EP, DeSantis TZ, Probst A, Andersen GL, Knight R, Hugenholtz P (2012). An improved Greengenes taxonomy with explicit ranks for ecological and evolutionary analyses of bacteria and archaea. ISME J.

[66] Yilmaz P, Parfrey LW, Yarza P, Gerken J, Pruesse E, Quast C, Schweer T, Peplies J, Ludwig W, Glockner FO (2014). The SILVA and “All-species Living Tree Project (LTP)” taxonomic frameworks. Nucleic Acids Res.

[67] Federhen S (2012). The NCBI Taxonomy database. Nucleic Acids Res.

[68] Zhang D, Xu S, Antwi P, Xiao L, Luo W, Liu Z, Li J, Su H, Lai C, Ayivi F (2019). Accelerated start-up, long-term performance and microbial community shifts within a novel upflow porous-plated anaerobic reactor treating nitrogen-rich wastewater via ANAMMOX process. RSC Advances.

[69] Oshiki M, Ali M, Shinyako-Hata K, Satoh H, Okabe S (2016). Hydroxylamine-dependent anaerobic ammonium oxidation (anammox) by “Candidatus Brocadia sinica”. Environ Microbiol.

[70] Lipsewers YA, Bale NJ, Hopmans EC, Schouten S, Sinninghe Damste JS, Villanueva L (2014). Seasonality and depth distribution of the abundance and activity of ammonia oxidizing microorganisms in marine coastal sediments (North Sea). Front Microbiol.

[71] Speth DR, Russ L, Kartal B, Op den Camp HJ, Dutilh BE, Jetten MS (2015). Draft genome sequence of anammox bacterium “Candidatus Scalindua brodae,” obtained using differential coverage binning of sequencing data from two reactor enrichments. Genome Announc.

[72] Yang Y, Li M, Li X-Y, Gu J-D (2018). Two identical copies of the hydrazine synthase gene clusters found in the genomes of anammox bacteria. Int Biodeterioration Biodegradation.

[73] Yang Y, Li M, Li H, Li XY, Lin JG, Denecke M, Gu JD (2020). Specific and effective detection of anammox bacteria using PCR primers targeting the 16S rRNA gene and functional genes. Sci Total Environ.

[74] Du R, Peng Y, Ji J, Shi L, Gao R, Li X (2019). Partial denitrification providing nitrite: opportunities of extending application for anammox. Environ Int.

[75] Hu Z, Wessels H, van Alen T, Jetten MSM, Kartal B (2019). Nitric oxide-dependent anaerobic ammonium oxidation. Nat Commun.

[76] Yang Y, Pan J, Zhou Z, Wu J, Liu Y, Lin JG, Hong Y, Li X, Li M, Gu JD (2020). Complex microbial nitrogen-cycling networks in three distinct anammox-inoculated wastewater treatment systems. Water Res.

[77] Parks DH, Rinke C, Chuvochina M, Chaumeil PA, Woodcroft BJ, Evans PN, Hugenholtz P, Tyson GW (2017). Recovery of nearly 8,000 metagenome-assembled genomes substantially expands the tree of life. Nat Microbiol.

