# Supplementary material for: High-quality bacterial genomes of a partial-nitritation/anammox system by an iterative hybrid assembly method
Source: Microbiome. 2020 Nov 6;8:155. doi: 10.1186/s40168-020-00937-3 (PMC7648391; doi:10.1186/s40168-020-00937-3)
Supplement: Supplementary file 2 — Additional file 1: Figure S1. Length distribution of long reads (LRs) generated on Oxford Nanopore PromethION platform. Figure S2. Initial binning results of PNA dataset using various short reads and long reads combination strategies. The short reads (SRs) and long reads (LRs) were hybrid assembled by Unicycler with parameter --min_fasta_length 1000 and then was binned by MetaWRAP (‘initial binning’ and ‘bin refinement’ modules with parameter -x 70 -c 10). Figure S3. Mismatches and indels rates of four assemblers, evaluated using PNA-with-Spiked-Mock dataset. (A), mismatches rate (B), indels rate. Orange triangle and red dashed line stand for the average and median value of the MAGs misassembly, respectively. The circle with red asterisk stands for a genome with mismatch rate more than 1000/Mbp. Figure S4. Assembler performance evaluation using Mock dataset, in which only contained a pool of SRs and LRs from mock community. Recovered MAGs number of different assemblers: megahit (6), metaspades (5), OPERA-MS (8), Unicycler (7). (A), Genomes recovery (B), Genome fraction vs genome purity (C), Genome continuity (D), Misassembly event occurred in assembled MAGs (E), Mismatches rates (F), Indels rates (G), gene recovery ratio. Figure S5. Assembler performance evaluation using the HMP dataset. Three cycles were performed using Unicyler-based IHA method. Only MAGs meeting the criteria (completeness > 70% and contamination < 10%) have been kept for downstream evaluation. (A-I) different evaluation indicators. Numbers in the legend indicate the MAG number retrieved using different assemblers. For B, the NGA50 improvement in each MAG pair was calculated by using NGA50 of the MAG obtained from IHA approach compared with megahit and metaSPAdes. Figure S6. Assembler performance evaluation using the GIS20 dataset. Three cycles were performed using Unicyler-based IHA method. Only MAGs meeting the criteria (completeness > 70% and contamination < 10%) have been kept for downstream e [file 40168_2020_937_MOESM1_ESM.docx]

**Supplementary materials for**

**High-Quality Bacterial Genomes of a Partial-Nitritation/Anammox System by an Iterative Hybrid Assembly Method**

Lei Liu^1,2,3^, Yulin Wang^1^, You Che^1^, Yiqiang Chen^1^, Yu Xia^1,2,3^, Ruibang Luo^4^, Suk Hang Cheng^5^, Chunmiao Zheng^2,3*^, Tong Zhang^1,3*^

Lei Liu: ^1^Environmental Microbiome Engineering and Biotechnology Laboratory, The University of Hong Kong, Hong Kong SAR, China. ^2^State Environmental Protection Laboratory of Integrated Surface Water-Groundwater Pollution Control, School of Environmental Science and Engineering, Southern University of Science and Technology, Shenzhen, China. ^3^School of Environmental Science and Engineering, Southern University of Science and Technology, Shenzhen, China. [liulei94@foxmail.com](mailto:liulei94@foxmail.com)

Yulin Wang: ^1^Environmental Microbiome Engineering and Biotechnology Laboratory, The University of Hong Kong, Hong Kong SAR, China.[yulinwang605@gmail.com](mailto:yulinwang605@gmail.com)

You Che: ^1^Environmental Microbiome Engineering and Biotechnology Laboratory, The University of Hong Kong, Hong Kong SAR, China. [nkcheyou@163.com](mailto:nkcheyou@163.com)

Yiqiang Chen: ^1^Environmental Microbiome Engineering and Biotechnology Laboratory, The University of Hong Kong, Hong Kong SAR, China. [chenyqnju@hotmail.com](mailto:chenyqnju@hotmail.com)

Yu Xia: ^1^Environmental Microbiome Engineering and Biotechnology Laboratory, The University of Hong Kong, Hong Kong SAR, China. ^2^State Environmental Protection Laboratory of Integrated Surface Water-Groundwater Pollution Control, School of Environmental Science and Engineering, Southern University of Science and Technology, Shenzhen, China. ^3^School of Environmental Science and Engineering, Southern University of Science and Technology, Shenzhen, China. [shuixia100@hotmail.com](mailto:shuixia100@hotmail.com)

Ruibang Luo: ^4^Department of Computer Science, The University of Hong Kong, Hong Kong SAR, China. [luoruibang@hku.hk](mailto:luoruibang@hku.hk)

Suk Hang Cheng: ^5^Department of Chemical Pathology, The Chinese University of Hong Kong, Hong Kong SAR, China. wonderland@cuhk.edu.hk

Chunmiao Zheng^*^ (Corresponding author): ^2^State Environmental Protection Laboratory of Integrated Surface Water-Groundwater Pollution Control, School of Environmental Science and Engineering, Southern University of Science and Technology, Shenzhen, China. ^3^School of Environmental Science and Engineering, Southern University of Science and Technology, Shenzhen, China. zhengcm@sustech.edu.cn

Tong Zhang^*^ (Corresponding author): ^1^Environmental Microbiome Engineering and Biotechnology Laboratory, The University of Hong Kong, Hong Kong SAR, China. ^3^School of Environmental Science and Engineering, Southern University of Science and Technology, Shenzhen, China. [zhangt@hku.hk](mailto:zhangt@hku.hk) ; Tel. 852-28578551; Fax 852-25595337.

**Figure legends**

**Figure S1**. Length distribution of long reads (LRs) generated on Oxford Nanopore PromethION platform.

**Figure S2**. Initial binning results of PNA dataset using various short reads and long reads combination strategies. The short reads (SRs) and long reads (LRs) were hybrid assembled by Unicycler with parameter --min_fasta_length 1000 and then was binned by MetaWRAP (‘initial binning’ and ‘bin refinement’ modules with parameter -x 70 -c 10).

**Figure S3**. Mismatches and indels rates of four assemblers, evaluated using PNA-with-Spiked-Mock dataset. (**A**), mismatches rate (**B**), indels rate. Orange triangle and red dashed line stand for the average and median value of the MAGs misassembly, respectively. The circle with red asterisk stands for a genome with mismatch rate more than 1000/Mbp.

**Figure S4**. Assembler performance evaluation using Mock dataset, in which only contained a pool of SRs and LRs from mock community. Recovered MAGs number of different assemblers: megahit (6), metaspades (5), OPERA-MS (8), Unicycler (7). (**A**), Genomes recovery (**B**), Genome fraction vs genome purity (**C**), Genome continuity (**D**), Misassembly event occurred in assembled MAGs (**E**), Mismatches rates (**F**), Indels rates (**G**), gene recovery ratio.

**Figure S5**. Assembler performance evaluation using the HMP dataset. Three cycles were performed using Unicyler-based IHA method. Only MAGs meeting the criteria (completeness > 70% and contamination < 10%) have been kept for downstream evaluation. (**A**-**I**) different evaluation indicators. Numbers in the legend indicate the MAG number retrieved using different assemblers. For B, the NGA50 improvement in each MAG pair was calculated by using NGA50 of the MAG obtained from IHA approach compared with megahit and metaSPAdes.

**Figure S6**. Assembler performance evaluation using the GIS20 dataset. Three cycles were performed using Unicyler-based IHA method. Only MAGs meeting the criteria (completeness > 70% and contamination < 10%) have been kept for downstream evaluation. (**A**-**I**) different evaluation indicators. Numbers in the legend indicate the MAG number retrieved using different assemblers. For B, the NGA50 improvement in each MAG pair was calculated by using NGA50 of the MAG obtained from IHA approach compared with megahit and metaSPAdes.

Figure S7. Initial binning result of PNA dataset of using 6 Gbp short reads (SRs) and 22 Gbp long reds (LRs). The figure was visualized by Bandage (v0.8.1). The number indicates the contig name, the blank circle stands for the single-contig MAG and the circle with colors means the circular sing-contig MAG.

**Figure S8**. Weighted functional gene sets distribution in 22 MAG pairs. Red asterisk stands for the genes in Set II and Set III were accounted more than 3% of total functional genes of the representative genome.

File S1. Genome information of 49 MAGs reconstructed by using IHA workflow (See Additional file 2: File S1)

File S2. Comparison of 26 MAG pairs reconstructed using hybrid and short reads only assembly approaches. (See Additional file 2: File S2)

File S3 Nitrogen related marker genes in the PNA system. (See Additional file 2: File S3)


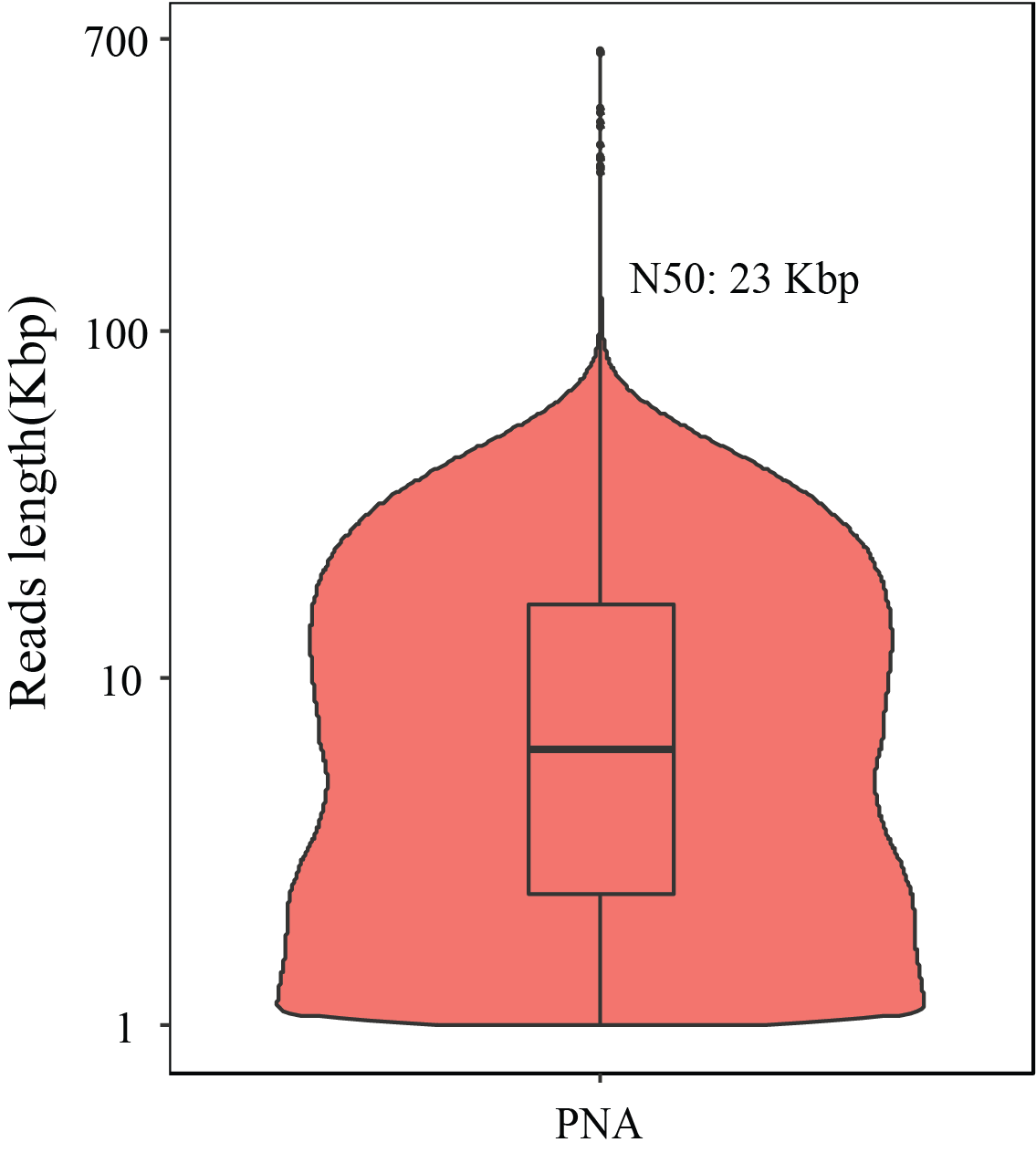


**Figure S1**. Length distribution of long reads (LRs) generated on Oxford Nanopore PromethION platform.


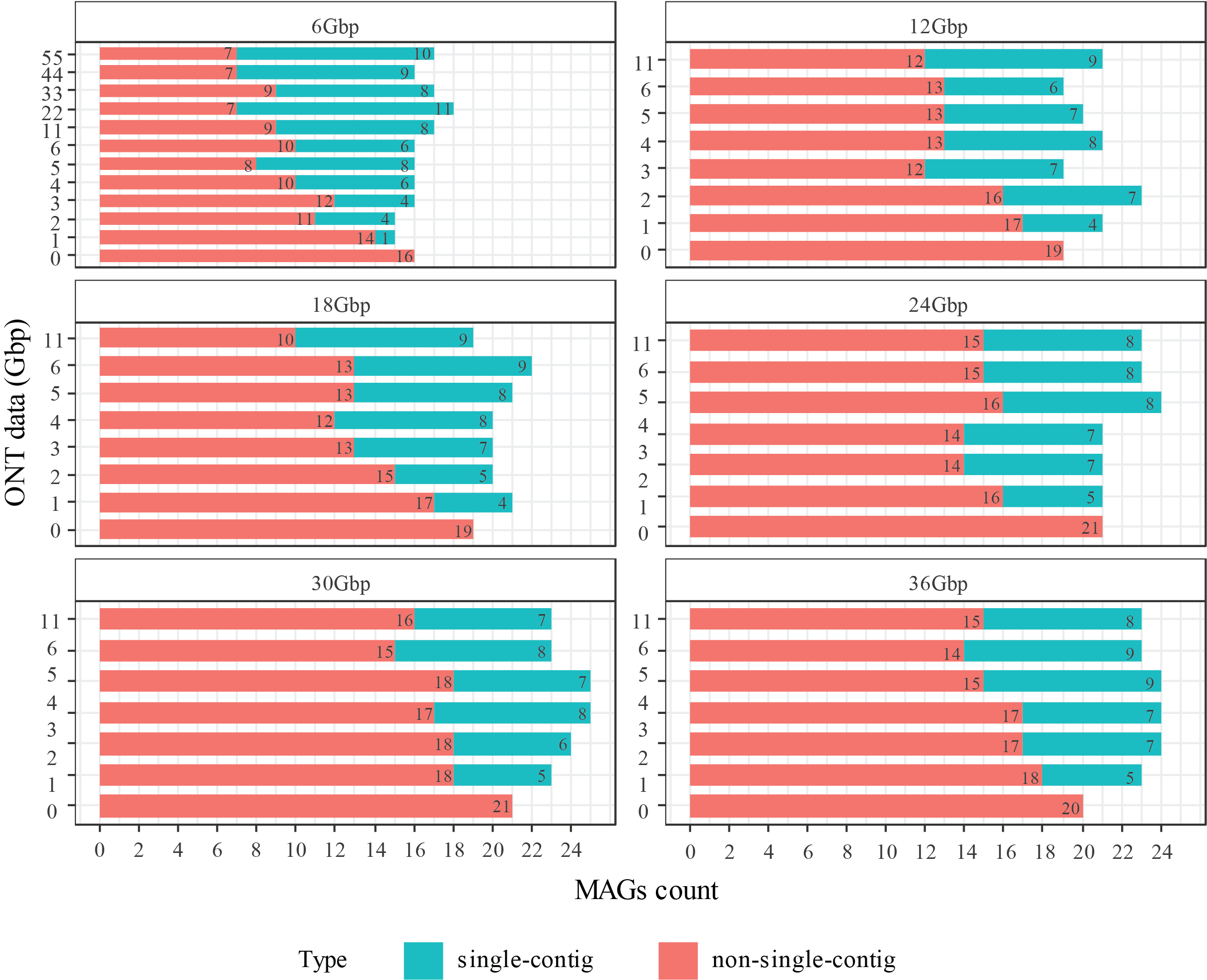


**Figure S2**. Initial binning results of PNA dataset using various short reads and long reads combination strategies. The short reads (SRs) and long reads (LRs) were hybrid assembled by Unicycler with parameter --min_fasta_length 1000 and then was binned by MetaWRAP (‘initial binning’ and ‘bin refinement’ modules with parameter -x 70 -c 10).


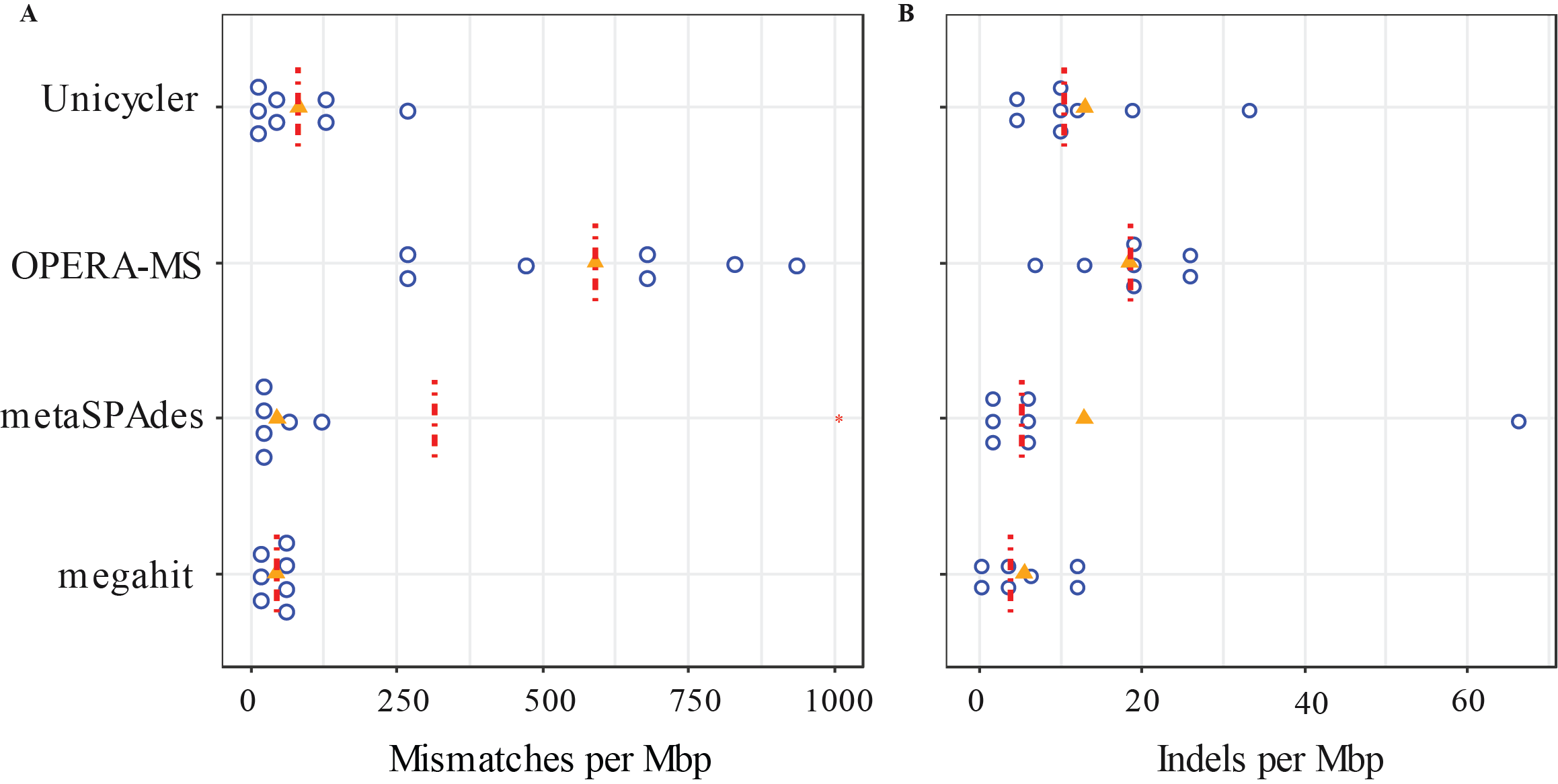


**Figure S3**. Mismatches and indels rates of four assemblers, evaluated using PNA-with-Spiked-Mock dataset. (**A**), mismatches rate (**B**), indels rate. Orange triangle and red dashed line stand for the average and median value of the MAGs misassembly, respectively. The circle with red asterisk stands for a genome with mismatch rate more than 1000/Mbp.


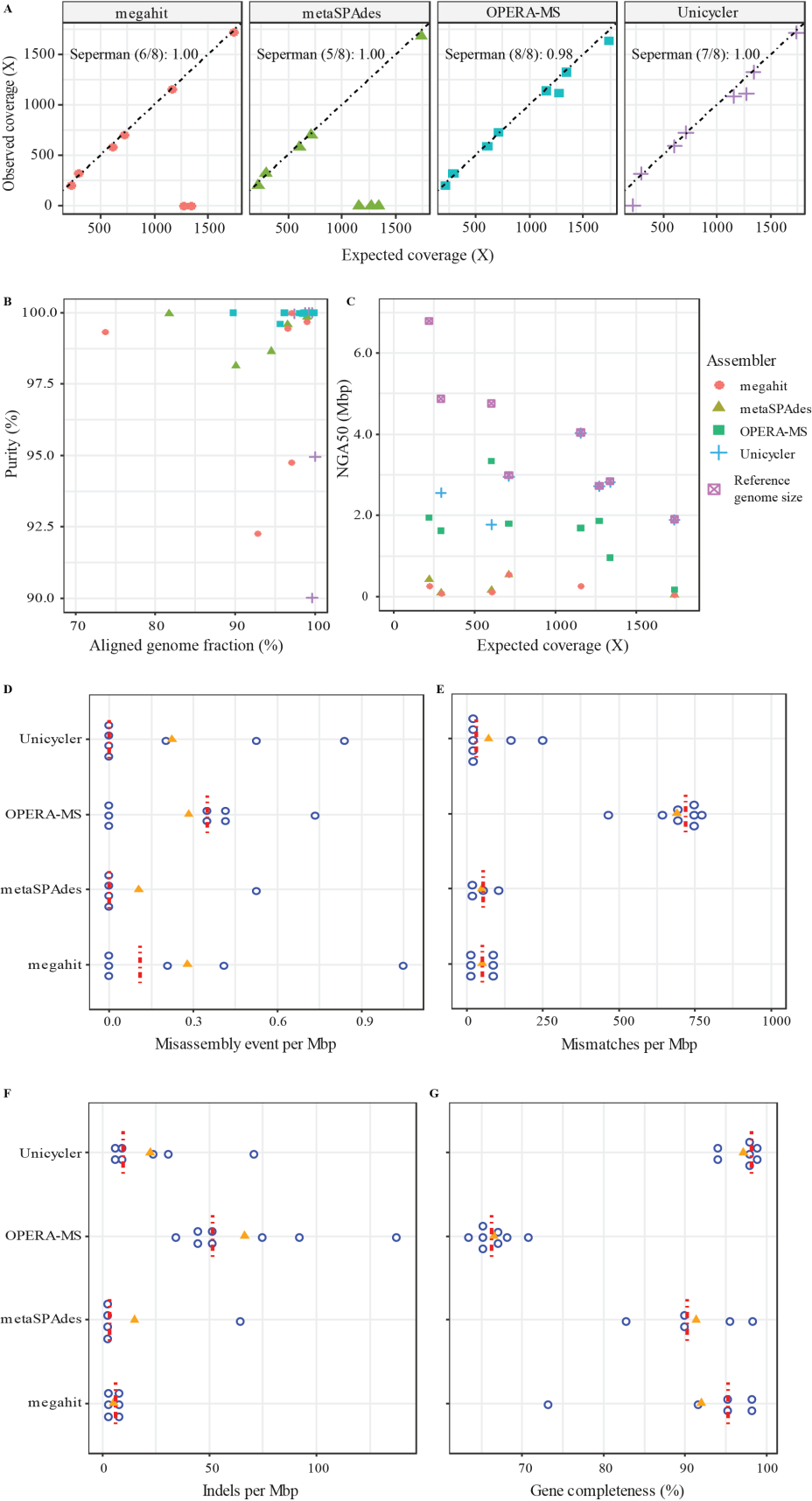


**Figure S4**. Assembler performance evaluation using the Mock dataset, in which only contained a pool of SRs and LRs from mock community. Recovered MAGs number of different assemblers: megahit (6), metaspades (5), OPERA-MS (8), Unicycler (7). (**A**), Genomes recovery (**B**), Genome fraction vs genome purity (**C**), Genome continuity (**D**), Misassembly event occurred in assembled MAGs (**E**), Mismatches rates (**F**), Indels rates (**G**), gene recovery ratio.


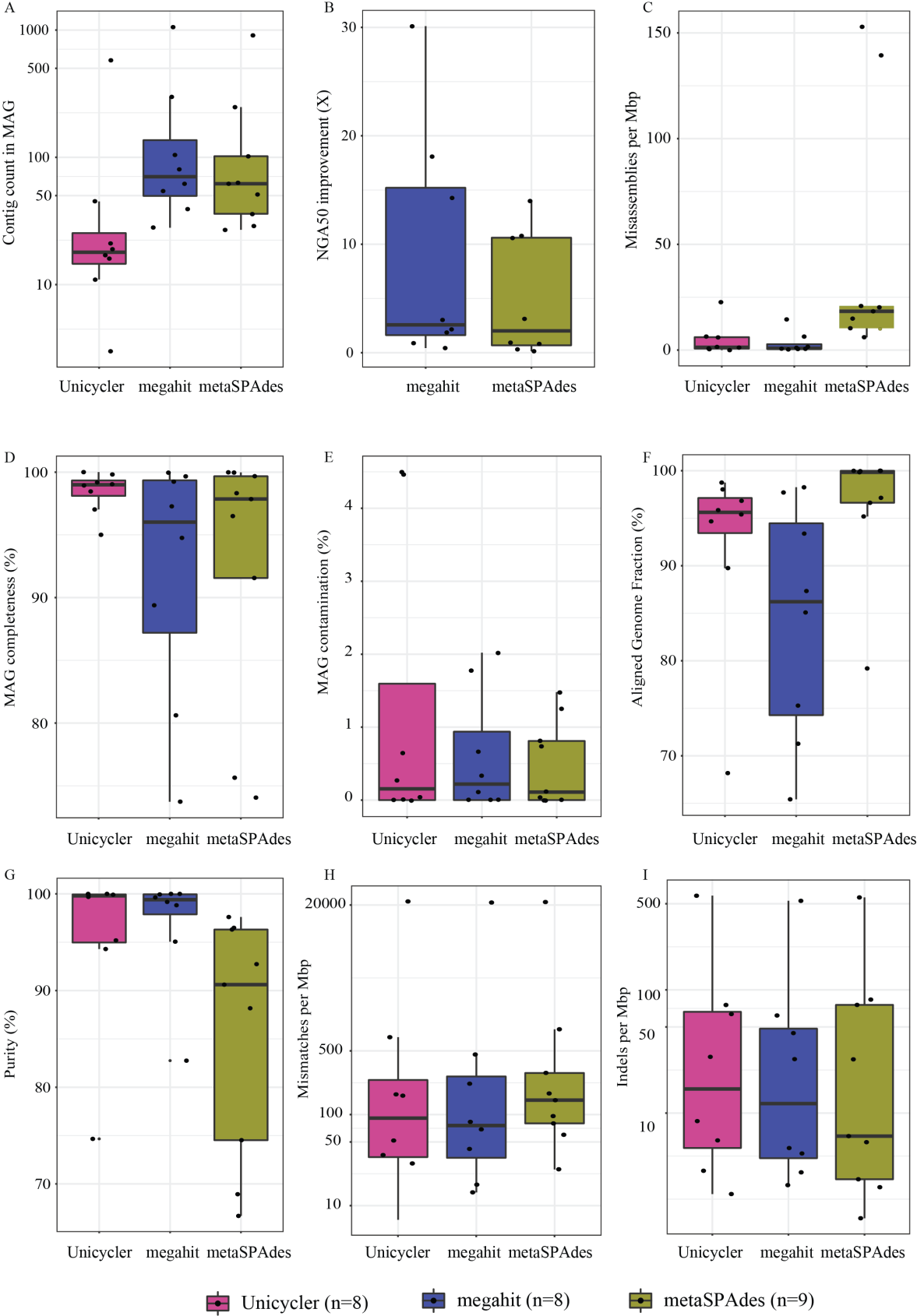


**Figure S5**. Assembler performance evaluation using the HMP dataset. Three cycles were performed using Unicyler-based IHA method. Only MAGs meeting the criteria (completeness > 70% and contamination < 10%) have been kept for downstream evaluation. (**A**-**I**) different evaluation indicators. Numbers in the legend indicate the MAG number retrieved using different assemblers. For B, the NGA50 improvement in each MAG pair was calculated by using NGA50 of the MAG obtained from IHA approach compared with megahit and metaSPAdes.


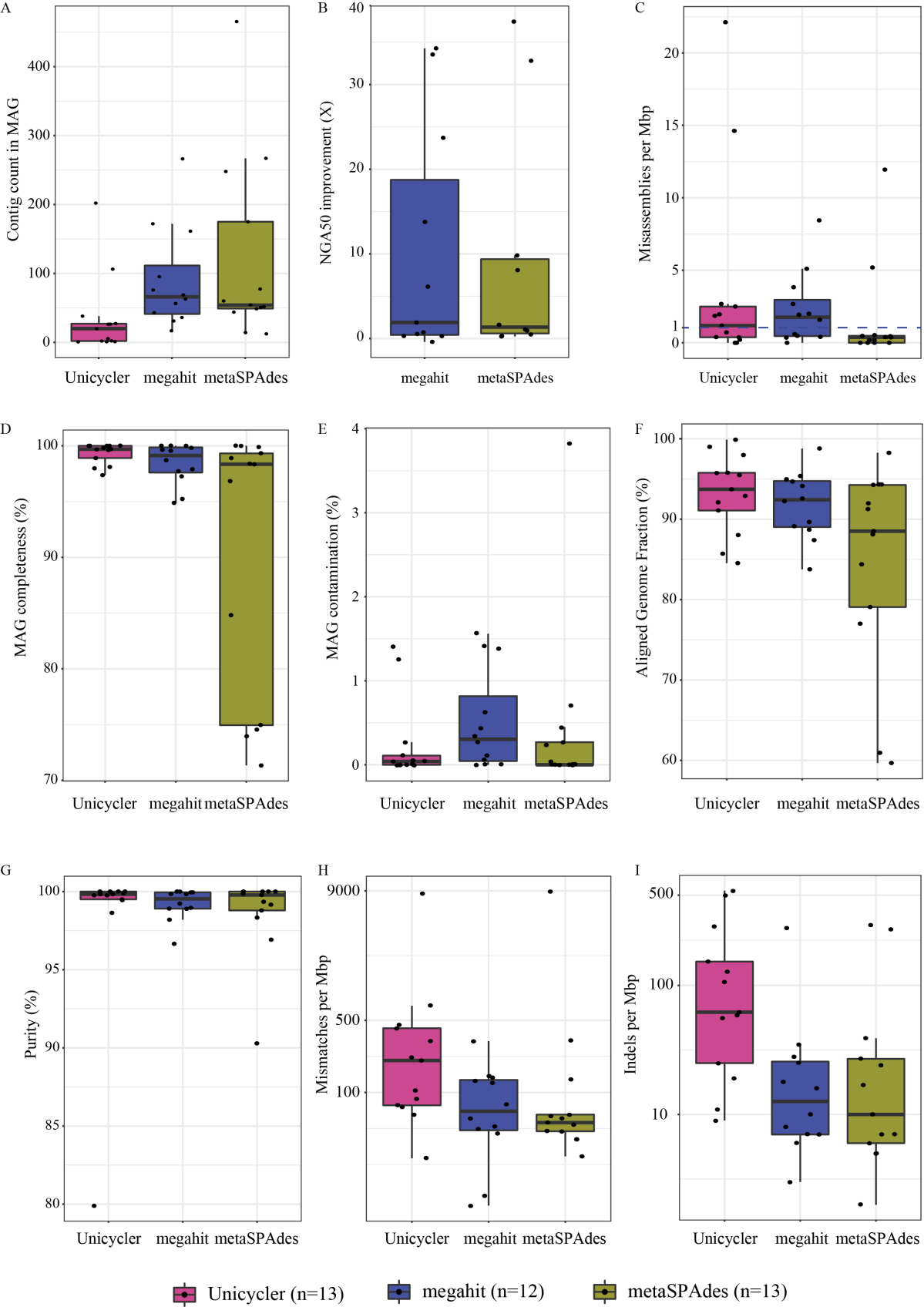


**Figure S6**. Assembler performance evaluation using the GIS20 dataset. Three cycles were performed using Unicyler-based IHA method. Only MAGs meeting the criteria (completeness > 70% and contamination < 10%) have been kept for downstream evaluation. (**A**-**I**) different evaluation indicators. Numbers in the legend indicate the MAG number retrieved using different assemblers. For B, the NGA50 improvement in each MAG pair was calculated by using NGA50 of the MAG obtained from IHA approach compared with megahit and metaSPAdes.


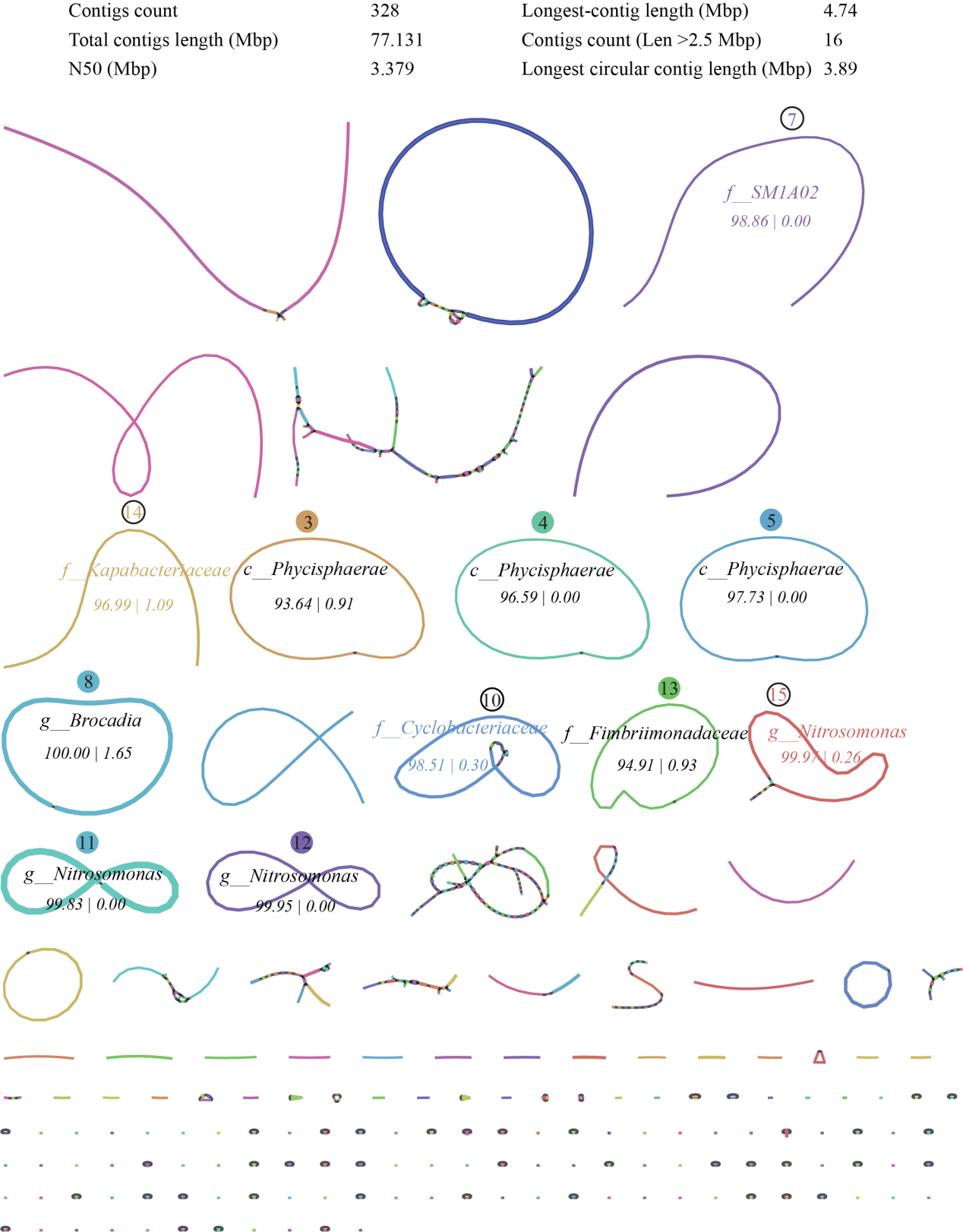


**Figure S7**. Initial binning result of PNA dataset of using 6 Gbp short reads (SRs) and 22 Gbp long reds (LRs). The figure was visualized by Bandage (v0.8.1). The number indicates the contig name, the blank circle stands for the single-contig MAG and the circle with colors means the circular sing-contig MAG.


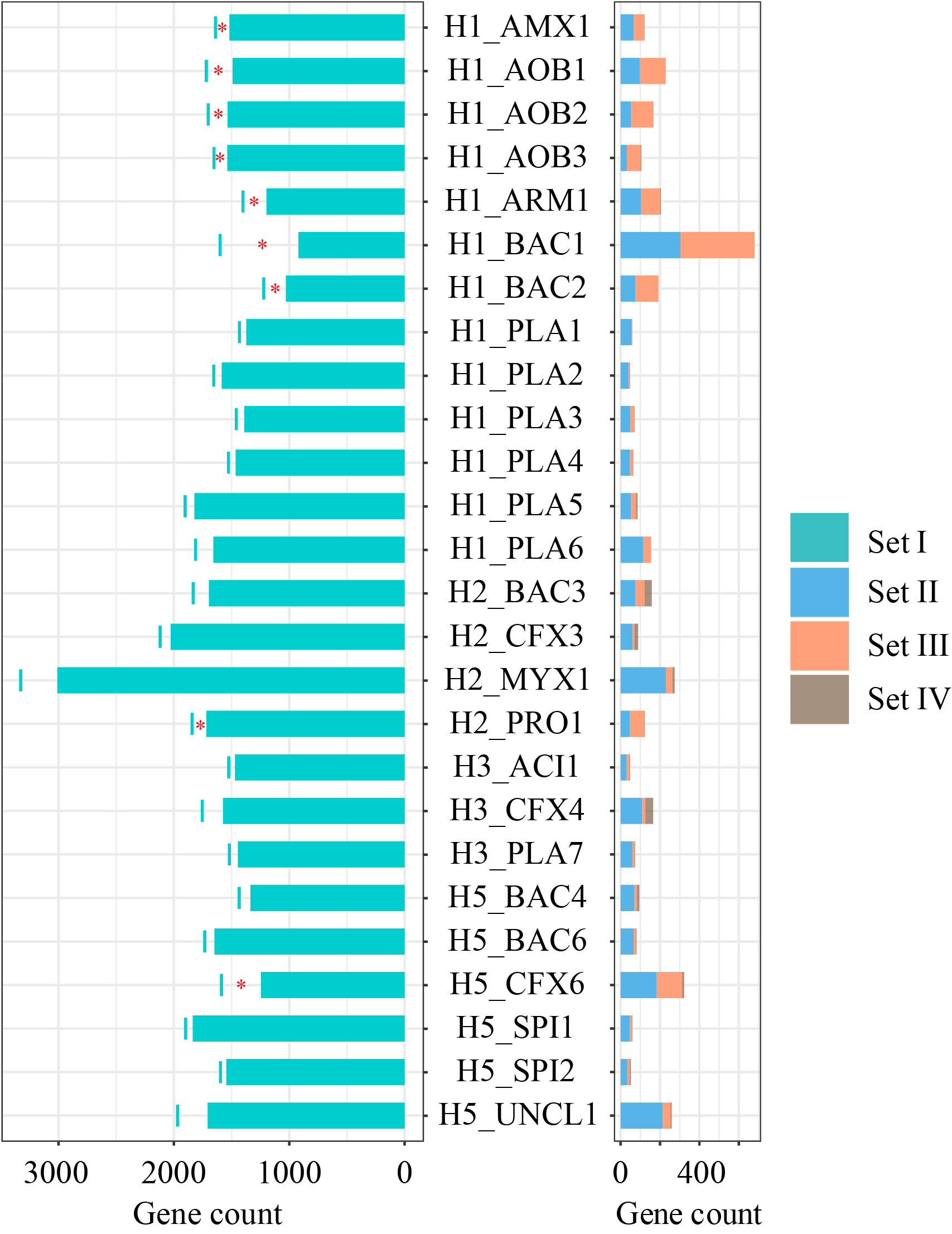


**Figure S8**. Weighted functional gene sets distribution in 22 MAG pairs. Red asterisk stands for the genes in Set II and Set III were accounted more than 3% of total functional genes of the representative genome.
